# Effect of a Nine-Month Web- and App-Based Workplace Intervention to Promote Healthy Lifestyle and Weight Loss for Employees in the Social Welfare and Health Care Sector: A Randomized Controlled Trial

**DOI:** 10.2196/jmir.6196

**Published:** 2017-04-10

**Authors:** Nina Charlotte Balk-Møller, Sanne Kellebjerg Poulsen, Thomas Meinert Larsen

**Affiliations:** ^1^ Department of Nutrition, Exercise and Sports Faculty of Science Copenhagen University Frederiksberg C Denmark

**Keywords:** health promotion, workplace, smartphone, weight reduction programs, Internet, eHealth, randomized controlled trial

## Abstract

**Background:**

General health promoting campaigns are often not targeted at the people who need them the most. Web- and app-based tools are a new way to reach, motivate, and help people with poor health status.

**Objective:**

The aim of our study was to test a Web- and mobile app-based tool (“SoSu-life”) on employees in the social welfare and health care sector in Denmark.

**Methods:**

A randomized controlled trial was carried out as a workplace intervention. The tool was designed to help users make healthy lifestyle changes such as losing weight, exercise more, and quit smoking. A team competition between the participating workplaces took place during the first 16 weeks of the intervention. Twenty nursing homes for elderly people in 6 municipalities in Denmark participated in the study. The employees at the nursing homes were randomized either 1:1 or 2:1 on a municipality level to use the SoSu-life tool or to serve as a control group with no intervention. All participants underwent baseline measurements including body weight, waist circumference, body fat percentage, blood pressure, and blood cholesterol level and they filled in a questionnaire covering various aspects of health. The participants were measured again after 16 and 38 weeks.

**Results:**

A total of 566 (SoSu-life: n=355, control: n=211) participants were included in the study. At 16 weeks there were 369 participants still in the study (SoSu-life: n=227, control: n=142) and 269 participants completed the 38 week intervention (SoSu-life: n=152, control: n=117). At 38 weeks, the SoSu-life group had a larger decrease in body weight (−1.01 kg, *P*=.03), body fat percentage (−0.8%, *P*=.03), and waist circumference (−1.8 cm, *P*=.007) compared with the control group.

**Conclusions:**

The SoSu-life Web- and app-based tool had a modest yet beneficial effect on body weight and body fat percentage in the health care sector staff.

**Trial Registration:**

Clinicaltrials.gov NCT02438059; http://clinicaltrials.gov/ct2/show/NCT02438059 (Archived by WebCite at http://www.webcitation.org/6i6y4p2AS)

## Introduction

### Background

Public health promoting campaigns have trouble reaching the socioeconomically disadvantaged groups [1] who really need them, and most face-to-face or telephone based interventions are generally considered too costly. Disadvantaged groups often need more targeted effort in order to be able to succeed in lifestyle changes. Therefore, Web- and app-based tools have been suggested as a new and potentially cost-effective way to reach out to, motivate, and help people to improve their health [2].

Web- and app-based tools for health promotion have been shown to have potential for weight loss programs and improving other health related factors. The number of these digital tools for health promotion being developed is increasing. The aim is generally to help users improve lifestyle and develop healthier habits, and thereby improve their health status. The need for effective tools for promoting health is evident from the recent report from the World Health Organization (WHO), which shows that overall health status in Europe is improving, but that the variation in health status is increasing both within and between countries [3].

In the Danish population, overall health, and in particular the distribution of overweight and obesity, is unequally distributed, with higher levels of overweight and obesity occurring among individuals with lower educational level [4]. Employees in the social and health sector generally belong to this group. A large number of these employees are organized in the trade union of public employees (FOA), where about 90% of the members are women. A recent health examination among 1737 female members of FOA, 59% of whom were women employed in the Danish social welfare and health care sector [5], found an increased prevalence of heavy smokers, overweight and obesity, and long-term sick leave compared with other employees at the same income level. However, the majority of the employees considered it very important that they maintain or improve their health [5].

Weight management is important for alleviating related health problems such as cardiovascular disease and type 2 diabetes in overweight individuals [3]. Overweight and obesity are not only associated with somatic diseases, but they are also related to several psychosocial complications, such as depression and anxiety [6,7], and to increased sick leave [8]. Furthermore, overweight, heavy smoking, and increased prevalence of long-term sick leave are significant precursors of chronic illnesses and early retirement [5,8,9]. The increasing prevalence of overweight and obesity, the associated health risks, and the increasing expenses related to these conditions make new and better targeted, well documented, and cost-effective health promotion programs for lifestyle change even more necessary.

The workplace is increasingly used as an arena for health promotion because it is the place where many people spend a great part of their time, and WHO has recommended that the workplace be prioritized as such an arena [10].

Web-based health promoting programs have occasionally been shown to assist weight loss in overweight and obese individuals. However, results are generally inconsistent and depend greatly on the type of Web-based tool used, as well as on the study design [11]. It is therefore intrinsically difficult to compare the results of different intervention studies using Web-based weight loss tools. However, a recent meta-analysis including 23 studies comprising 8697 participants found that Web-based tools for weight loss had a modest but significant effect on weight loss: −0.68 kg compared with a nontreated control group [12]. A Cochrane analysis found an effect of −1.5 kg after 6 months [13].

The effect of workplace health promotion interventions has generally been found to be rather small and greatly dependent on the quality of the study [14]. Only a few Web- and app-based intervention studies have been conducted in workplace settings [15-17], and only two of these were randomized controlled trials [16,17]. A study by Van Vier et al [16] found that a Web-based intervention could result in a weight loss of −1.1 kg and a reduction in waist circumference of −1.2 cm, and a study by Cook et al [17] found that a Web-based intervention was more effective than printed material in inducing improvements in diet and nutrition, though this study did not examine changes in body weight.

Mobile phone apps may be useful as health promoting tools. A systematic review with meta-analysis including 17 studies with the use of mobile devices for weight loss found that the use of mobile devices induced weight loss [18]. A recent study investigating the potential of a Web-based app for promoting a healthy lifestyle found promising results [19]. However, the field has not been sufficiently explored and the potential of mobile phone apps for health promotion is not fully understood [20]. There is evidence that adding a social feature to the intervention, such as a team-based element where users can compare and compete with each other, can have a positive effect on users’ willingness to use a mobile app for health promotion [21]. We have found no previous studies that have combined the use of a workplace Web-based health promoting program with a social feature.

### Aim of This Study

The primary aim of this study was therefore to investigate the effect of the SoSu-life Web- and mobile phone-based app, in combination with a social feature, on changes in body weight. Secondary outcomes were changes in body fat percentage, waist circumference, blood pressure and total cholesterol (all reported in this paper), behavior change, and self-perceived well-being (both reported in a paper under preparation). User-data and qualitative data from the study are reported in another paper (Balk-Møller, under review in JMIR 2017). The control group underwent the same examinations as the intervention group, which provided information about the stand-alone effect of such health examinations.

## Methods

### Sampling and Study Design

**Figure 1:**
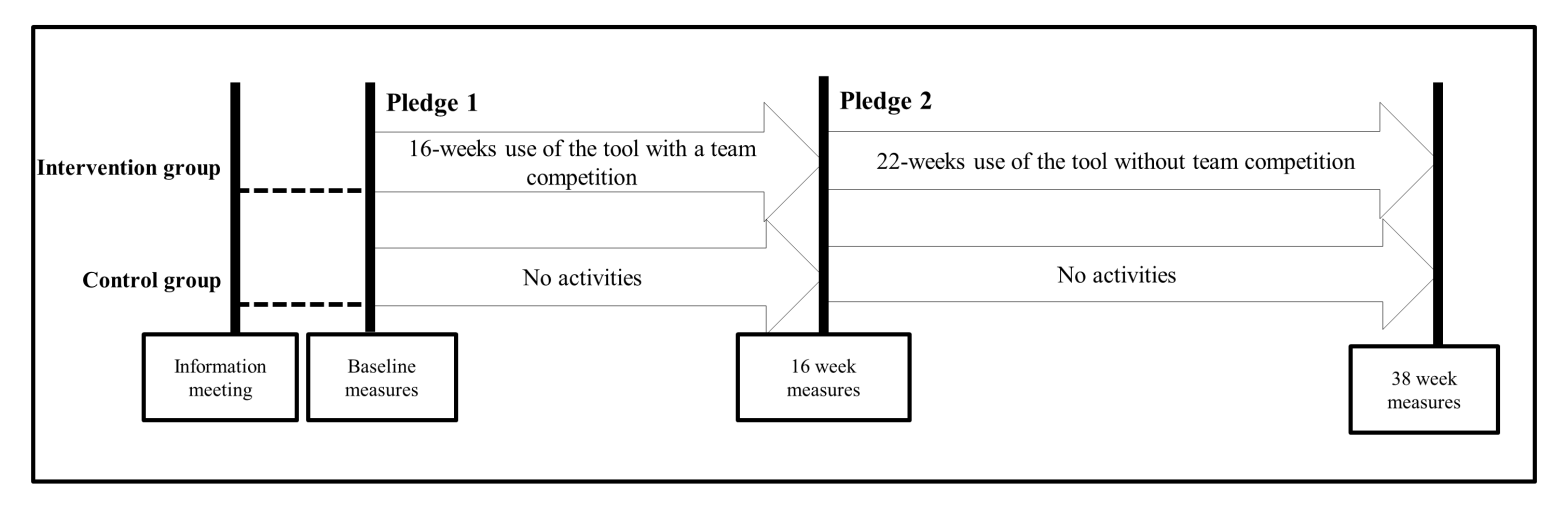
Overview of the SoSu-life study.

The study was conducted in 6 municipalities in Denmark from August 2012 to July 2013. In each municipality, 2-4 nursing homes were randomized to either the SoSu-life tool (called SoSu-life group) or to a control group. The randomization of the participating nursing homes was conducted continually over time within each municipality. Those municipalities with an even number of nursing homes were randomized in a 1:1 ratio and municipalities with an odd number of nursing homes in a 2:1 ratio. Of the total 20 nursing homes, 12 were randomized to SoSu-life and 8 were randomized to control. The randomization was performed in a simple blinded way (simple paper draw) by 2 of the study investigators in a collaboration between investigators and staff at an initial meeting with local staff in each municipality. Each draw was observed by independent witnesses to observe that it was performed in a fair and unbiased way. The study was divided into two distinct periods for the SoSu-life group: an initial 16-week period including a team competition, and a subsequent 22-week period without a competition. Participants chose a pledge (described later) for each period (Figure 1).

Participants were recruited from August 2012 to September 2012 when an information meeting was held at each nursing home during work hours, after which each of the interested employees completed the informed consent form. Eligibility criteria for participation were that the employees had to work under conditions in accordance with a FOA-negotiated agreement. Computer or mobile app literacy was not an inclusion criterion. A few weeks later, the participants underwent a baseline clinical examination conducted by trained staff from the research group. The clinical assessments were all performed locally at each participating nursing home, which made it impossible for the research staff to be blinded to the participants’ group allocation. The anthropometrical assessments were measured using a wall-mounted stadiometer for height and a digital electronic scale (Tanita WB 100MA/WB-110MA III) for body weight. Measuring tape was used for measuring hip and waist circumference taken at the widest part of the hip and at the umbilicus, respectively. Body fat percentage was recorded with a handheld body composition monitor (Omron BF306) and blood pressure was measured using a digital blood pressure manometer (Kivex, Automatic Blood Pressure Monitor, Model UA-787 Plus). Finger-prick blood samples were drawn to measure total cholesterol (Accutrend Plus; more information in Multimedia Appendix 1). Participants in the intervention group signed up for their first pledge (Pledge 1) immediately after the clinical examination, and were then introduced to the SoSu-life website and app by a member from the project team, and given a pamphlet about the content and functionalities of the tool. This session took approximately 30-45 min. The clinical examination, taking about 15 min in total, was repeated at 16 weeks (December 2012 to January 2013). The participants in the intervention group then made a new pledge (Pledge 2). The examination was repeated at 38 weeks (May 2013 to June 2013), when participants also answered a set of questions addressing their overall evaluation of the study. The participants in both groups spent approximately 10-15 min answering a Web-based questionnaire based on a questionnaire developed by the Danish National Research Centre for the Working Environment, regarding demographic data, health behavior, and general well-being the day before each of the 3 clinical examinations. The study was approved by the ethical committee of the Capital Region of Denmark (reference number: H-2-2012-079; a more detailed description of the study design is given in the Multimedia Appendix 1). The study is registered at Clinicaltrials.gov with ID number NCT02438059.

### Developing the Tool

The SoSu-life tool is a Web- and app-based tool developed specifically for this particular group of employees. It was created in a collaboration between the company Mobile Fitness, employees from the sponsoring company PenSam, who work with health promotion for the social and health care workers on a daily basis, and scientists from the University of Copenhagen. The tool was developed through an iterative process where the users (ie, the social and health care workers) and the developers (Mobile Fitness, PenSam, and Copenhagen University) were involved. Initially, focus groups from the target group were held, where ideas and mock-ups were presented. The first edition of the SoSu-life tool was tested in a pilot study at two nonparticipating nursing homes. The ideas for mechanisms that could lead to behavior change were drawn from experience in earlier health promotion projects (nondigital) among social and health care workers, and from Mobile Fitness’ expertise with a digital weight loss tool used in a similar population, both with individual users and applied in workplace settings (unpublished data).

### Description of the SoSu-Life Tool

The tools’ basic features were self-reporting of diet and exercise, personalized feedback, suggestions for activities and programs, and practical tips and tricks. A series of social features, including weekly assignments and colleague challenges designed to create social support and build social interactions at the workplace, played a vital role in promoting behavior change. The SoSu-life tool aimed to mobilize whole groups of colleagues to not only encourage each other in achieving personal goals, but also work on identical small weekly assignments. Points were assigned to all individual and group activities and were collected for both individuals and groups. Individual activities gave points not only to the individual but also to the group as part of the group competition. In this way, each individual´s use of the digital tool benefited the whole group.

On entering the study, each participant chose one pledge out of seven to focus upon: lose weight, eat healthier, improve physical fitness, improve physical strength, quit smoking, decrease the number of cigarettes smoked, or maintain a healthy lifestyle. The program itself recommended a pledge based on information obtained from the health examination. The choice of pledge was designed to give the participants a key focus area. Subsequent communication—feedback, frequency, and content of emails and text messages provided by the program, and topics for user-to-user communication—was targeted according to the participants’ individual pledge. Messages contained information about specific health issues related to the pledge, general tips, and tricks on health and well-being.

The program had various tools to help the user succeed with the pledges. The self-reporting of diet and exercise was a weight loss tool based on a unit system designed to assist either weight loss or maintenance of a weight loss [22]. All foods were assigned a number of units based on portion size, calories, and macronutrient composition [22]. Daily energy level was calculated based on the users’ height and weight, and on this basis the number of units per day advisable for weight loss was suggested. The user registered his or her food intake and daily exercise, and the program gave feedback on the energy balance of the day, a green code indicating a proper energy balance, and a red code for excessive energy intake. Exercise was registered as bonus-units according to standardized energy expenditure equivalents related to specific types of exercise [23] (more information is given in the Multimedia Appendix 1). The same system was used for those participants wishing to focus on exercise alone, and feedback was given in the form of number of earned bonus-units. The website provided access to a number of video-supported exercise programs designed to increase fitness level or improve strength. Smokers wishing to either change their smoking habits or quit smoking were advised to begin by registering their habitual use of cigarettes, the time the cigarettes were smoked, and the mood they were in when they were smoking the cigarettes.

**Figure 2:**
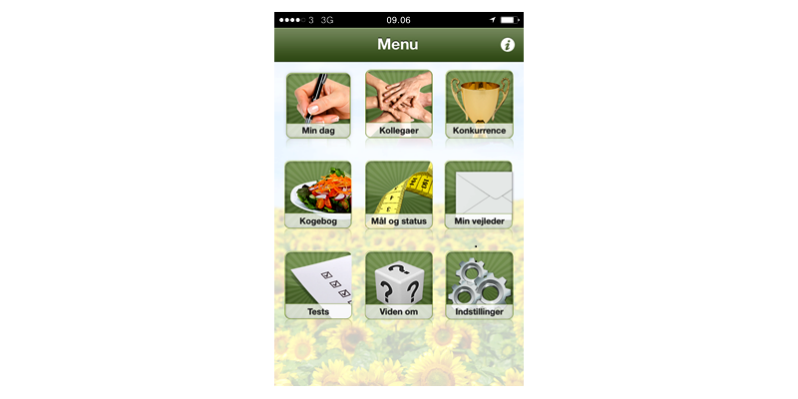
Screenshot of the SoSu-life app menu.

**Figure 3:**
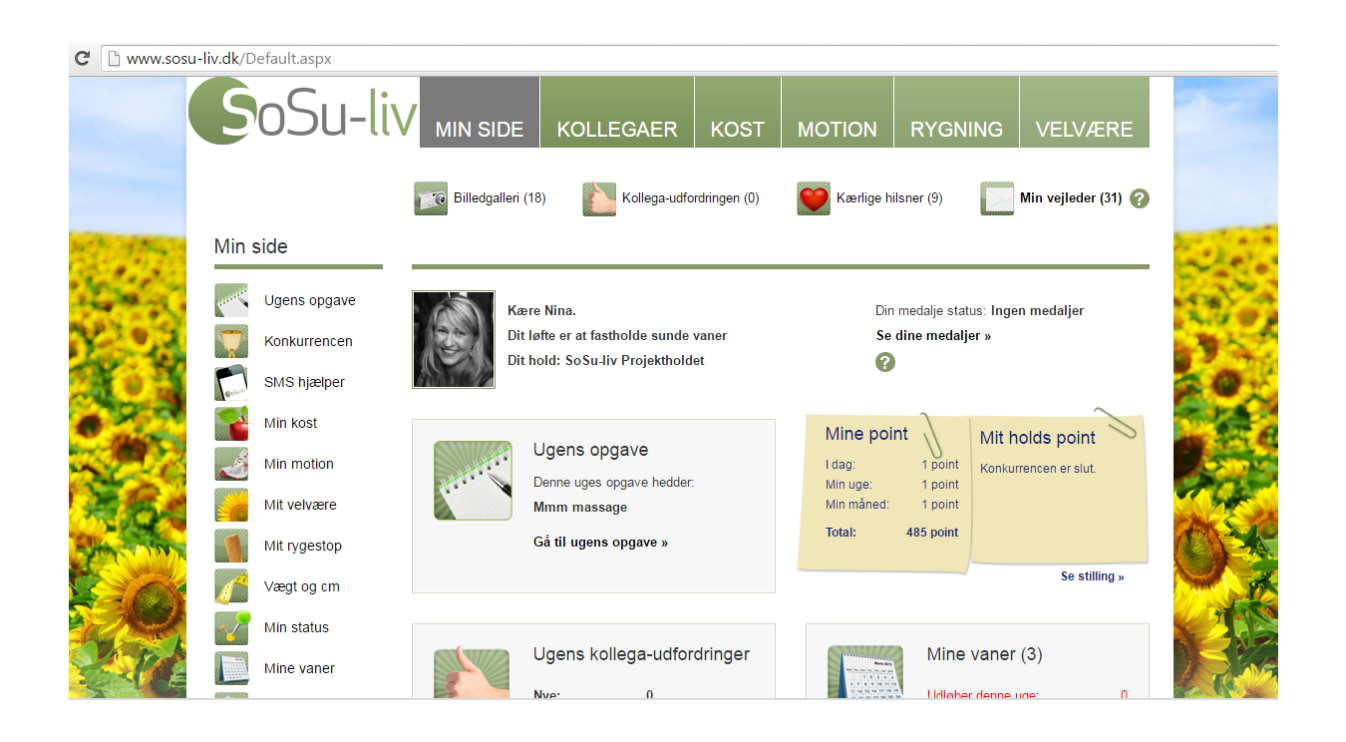
Screenshot of the SoSu-life website.

The social features included weekly assignments for all group members. Such weekly assignments could be “drink at least one liter of water every day all week” or “remember to say Good Morning to your colleagues every morning all week.” The tool also included “colleague challenges” that were to be sent from colleague to colleague and were determined by the participants’ individual pledge. Challenges might be: “Do not eat sugar for three days” or “Bring some fruit for us two to eat together tomorrow during the afternoon break.” All features could be accessed from both the app and website (Figures 2 and 3).

The SoSu-life program entails a team competition and used a point system where all activities performed using the tool gave points to the individual user. The point system provided the highest reward for taking part in social activities. Performing the weekly challenges, and sending and carrying out “colleague challenges,” were rewarded with more points than registering diet or exercise, or taking tests or quizzes. During the first 16 weeks, each of the participating nursing homes or home care units constituted a team, and each of the individual participants’ points were added to the teams’ total points. A lottery ticket was generated for each point earned by the team. Each month the teams had a chance to win a prize from a simple lottery drawn by the research staff. The more points the team had, the bigger the chances of winning. The prizes included things such as a shopping bag for each team member, a Zumba class for the team, or a visit from a bartender who served fruit smoothies during lunch hours. The team that collected the most points after 16 weeks also won a prize. Points were still collected in the second (22-week) intervention period, but no prizes were provided. The cost of the prizes was covered by the main sponsor of the project, who also presented the prizes at local celebration events at the nursing homes. The social features and the team competition were designed to create a supporting and encouraging work environment to help generate behavior change in the individual participant. A more complete description of the tool based on the TlDieR-checklist is given in the Multimedia Appendix 1. The content in the intervention remained the same during the 38 weeks, and there were no significant bug fixes or downtimes during the project.

### Statistical Analyses

All statistical analyses were performed in Stata release 11 (StataCorp LP). Differences in anthropometric measures from baseline to week 16, and from baseline to week 38, were analyzed using a multilevel mixed effect linear regression (mixed model) with municipality as a random effect. Significance was set at *P* value ≤.05 for all analyses.

Descriptive statistics of the sample were presented as mean and standard deviation (SD). Categorical data were presented as percentage of the sample (n). Differences between groups were tested with unpaired *t* test, and chi-squared for categorical data.

Use of the SoSu-life tool differed according to the pledge taken. Participants pledging change of smoking habits were therefore left out of the anthropometric outcomes. A separate analysis of a subgroup of participants with weight loss pledge, both during the first 16 weeks and the last 22 weeks (subgroup with weight loss pledge 1 and 2), was carried out.

The study was designed and powered as a “completers only” study, considering the completer analysis as the primary outcome, and that an intention-to-treat (ITT) analysis should serve as a sensitivity analysis. This was decided on the basis of two major considerations. First, the study was designed as a real-life intervention applied in a workplace setting, and participation was completely voluntary. The information provided to the study participants in the recruitment phase was relatively brief, approximating what we consider realistic if the intervention was to be disseminated on a much larger scale. Second, the pragmatic study design with only 4 visits at each workplace (information meeting, baseline meeting, week 16, and week 38) essentially gave no room for any rescheduling of clinical measures and completion of Web-based questionnaires at the workplaces. This meant that absence from work on the clinical visit days, whether due to time off or illness, automatically led to missing data.

We believe this would have resulted in quite a significant amount of missing data. However, when performing an ITT baseline carried forward analysis, the results showed no major changes (see Multimedia Appendix 1).

### Power Calculations

Change in body weight among study completers was used for the power calculations. Recruiting a total of 450 participants divided equally between the 2 study groups, and assuming (1) dropout rate of 25% and 20% over the 16 weeks and 22 weeks intervention periods, respectively, (2) a between-group difference of 2.0 kg, (3) SD of 4.0 kg in both groups, and (4) a significance level of .05, this would result in a power of 0.98. Fewer people volunteered to participate in and fewer completed the study than anticipated (152 in the intervention group vs 117 in the control group). A post hoc power analysis based on the actual results found a power of 0.27.

## Results

### Description of the Study Sample

**Figure 4:**
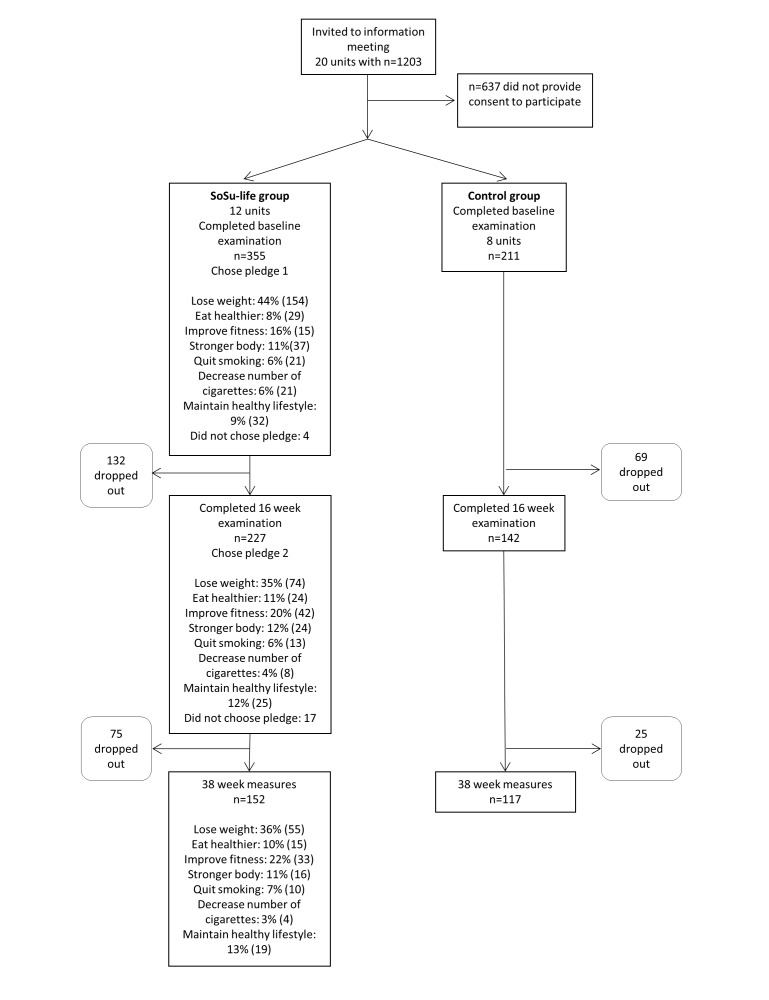
Flow diagram of the participants.

Initially, 1203 employees from 20 different nursing homes in 6 municipalities were invited to participate in the study. After the information meeting, a total of 566 participants signed the consent form and underwent baseline examination (n=355 in the intervention group, n=211 in the control group). The second examination took place after 16 weeks, when 65.2% (369/566) participants were measured (n=227 in the intervention group, n=147 in the control group). The same procedure was applied after the total of 38 weeks, when 47.5% (269/566) still remained in the study (n=152 in the intervention group, n=117 in the control group; Figure 4).

There were no significant differences at baseline between the groups regarding social and anthropometric characteristics (Table 1).

**Table 1:** Social and anthropometric characteristic at baseline for completers in SoSu-life group and control group.

Variable		SoSu-life^a^	Control^b^	*P* value
Age (in years), mean (SD^c^)		47.0 (10.0)	47.0 (9.9)	.99^d^
Females, n (%)		140 (92.1)	108 (92.3)	.95^e^
**Work schedule^f^, n (%)**				
	Day duty	118 (77.6)	90 (79)	.88^e^
	Evening duty	18 (11)	15 (13.2)	
	Night duty	8 (5)	4 (4)	
	Varying shifts	8 (5)	5 (4)	
**Marital status^g^, n (%)**				
	Single	47 (31)	31 (27)	.44^e^
	Cohabitate	103 (68.7)	84 (73)	
**Children in the household^h^, n (%)**				
	Yes, 1 child	36 (24)	39 (34)	.08^e^
	Yes, 2 children	31 (20)	14 (12)	
	Yes, 3 or more	15 (10)	16 (14)	
	No	70 (46)	45 (40)	
Height (cm), mean (SD)		166.2 (7)	165.5 (7.2)	.41^d^
Body weight (kg), mean (SD)		74.5 (15.9)	73.1 (14.8)	.44^d^
Waist circumference (cm)^i^, mean (SD)		92.8 (13.9)	91.2 (11.9)	.27^d^
Hip circumference (cm), mean (SD)		103.6 (10.8)	101.6 (10.8)	.12^d^
Total cholesterol (mmol/L)^j^, mean (SD)		5.3 (0.9)	5.3 (0.9)	.78^d^
Body fat percentage (%)^k^, mean (SD)		35.3 (7.4)	35.1 (7.4)	.88^d^
Systolic blood pressure (mmHg), mean (SD)		129.0 (16.5)	130.8 (16.4)	.37^d^
Diastolic blood pressure (mmHg), mean (SD)		80.9 (9.6)	82.1 (10.0)	.32^d^

^a^SoSu-life: n=152, unless other is specified.

^b^Control: n=117, unless other is specified.

^c^SD: standard deviation.

^d^Data are compared between groups using students *t* test.

^e^Data are compared between groups using chi-square test.

^f^Control: n=114.

^g^SoSu-life: n=150, control: n=115.

^h^Control: n=114.

^i^Control: n=116.

^j^SoSu-life: n=151, control: n=112.

^k^Body fat percentage device had a maximum of 49.9 %, SoSu-life: n=148, control: n=115.

Our study population is representative of the general characteristics of social and health care workers in Denmark [24]. The study included slightly more participants working day shifts (77%) than is normal in this sector (60%). The study population also included fewer shift workers (around 5% compared with 15% in general) [24]. Dropout rate was higher for shift workers and for evening workers. Dropouts were otherwise generally comparable with those that completed the study, except that they had a higher waist circumference (data shown in Multimedia Appendix 1).

### Changes in Body Weight and Anthropometric Markers

During the intervention period the SoSu-life group (excluding participants with pledges regarding smoking) had a larger decrease in body weight of −1.01 kg (*P*=.03), body fat percentage −0.78% (*P*=.03), and waist circumference of −1.79 cm (*P*=.007) after a total of 38 weeks compared with the control group. The weight loss subgroup had an even larger decrease in body weight of −1.64 kg (*P*=.02) and waist circumference of −2.47 cm (*P*=.008) after 38 weeks compared with the control group.

The SoSu-life group (excluding participants with pledges regarding smoking) had a larger decrease in body weight of −1.54 kg (*P*<.001) and a decrease in body fat percentage of −0.81% (*P*=.003) compared with the control group during the first 16 weeks. Here the weight loss subgroup had a decrease in body weight of −2.36 kg (*P*<.001), a decrease in body fat percentage of −0.99% (*P*=.003), and a decrease in waist circumference on −2.45 cm (*P*=.003) compared with the control group (Table 2).

No significant difference was observed between any of the SoSu-life groups or the control group in any of the other outcomes, such as blood pressure or total cholesterol.

When performing an ITT-analysis (results presented in Multimedia Appendix 1), as expected, the overall effect size between Sosu-Life and control group decreased, but remained statistically significantly different for the primary outcomes, such as body weight change. Hence, the ITT-analysis only differed from the “completers only” results in terms of the changes in body fat percentage from week 0 to week 38 between SoSu-life group and control that went from a significant (*P*=.03) to nonsignificant (*P*=.20), changes in waist circumference from week 0 to week 16 between SoSu-life group and control that went from nonsignificant (*P*=.09) to significant (*P*=.03), and from week 0 to week 38 between subgroup and control that went from significant (*P*=.008) to nonsignificant (*P*=.05).

**Table 2:** Changes in body weight, body fat percentage, waist circumference, systolic blood pressure, diastolic blood pressure, and total cholesterol from week 0 to week 16 and from week 0 to week 38 for completers in Web- and app-based intervention for health promotion.

Changes from week 0 to week 16 and from week 0 to week 38		n	SoSu-life Mean (SE^a^)	n	Subgroup with weight loss pledge^b^ Mean (SE)	n	Control Mean (SE)	Adjusted difference^c^ between SoSu-life group and control (95% CI)	*P* value^c^ between SoSu-life group and control	Adjusted difference between subgroup^b^ and control (95% CI)	*P* value^c^ between subgroup^b^ and control
Body weight^d^(kg)	week 0 to 16	130	−1.44 (0.26)	41	−2.39 (0.52)	117	0.10 (0.19)	−1.54 (−2.18 to −0.90)	<.001	−2.36 (−3.23 to −1.49)	<.001
	week 0-38	130	−1.04 (0.34)	41	−1.68 (0.77)	117	−0.03 (0.33)	−1.01 (−1.94 to −0.08)	.03	−1.64 (−3.04 to −0.24)	.02
Body fat Percentage^d^	week 0-16	127	−0.70 (0.21)	38	−0.97 (0.29)	114	0.04 (0.16)	−0.81 (−1.35 to −0.27)	.003	−0.99 (−1.63 to −0.34)	.003
	week 0-38	126	−1.50 (0.23)	37	−1.13 (0.40)	114	−0.71 (0.27)	−0.78 (−1.48 to −0.09)	.03	−0.39 (−1.43 to 0.64)	.46
Waist Circumference^d^ (cm)	week 0-16	130	−1.40 (0.47)	41	−3.12 (0.93)	116	−0.51 (0.37)	−1.05 (−2.26 to 0.16)	.09	−2.45 (−4.09 to −0.81)	.003
	week 0-38	129	−0.67 (0.50)	40	−1.90 (0.96)	116	−0.04 (0.44)	−1.79 (−3.09 to −0.49)	.007	−2.47 (−4.30 to −0.63)	.008
Systolic blood pressure (mmHg)	week 0-16	152	−2.27 (0.93)	41	−1.90 (1.43)	117	−2.57 (1.03)	0.30 (−2.41 to 3.02)	.83	−0.52 (−3.42 to 2.37)	.66
	week 0-38	152	−2.82 (1.06)	41	−2.93 (1.56)	116	−5.83 (1.22)	2.27 (−0.96 to 5.50)	.17	−0.63 (−3.42 to 2.16)	.72
Diastolic blood pressure (mmHg)	week 0-16	152	−1.85 (0.64)	41	−1.71 (2.02)	117	−1.48 (0.72)	−0.57 (−2.48 to 1.35)	.56	0.87 (−3.23 to 4.96)	.68
	week 0-38	152	−2.02 (0.62)	41	−4.02 (2.24)	117	−2.30 (0.65)	0.10 (−1.69 to 1.90)	.91	1.37 (−3.43 to 6.16)	.58
Total cholesterol (mmol/L)	week 0-16	149	0.10 (0.07)	40	0.20 (0.12)	112	0.20 (0.09)	−0.09 (−0.30 to 0.13)	.43	−0.01 (−0.33 to 0.31)	.97
	week 0-38	148	−0.12 (0.07)	40	−0.13 (0.17)	111	0.01 (0.08)	−0.10 (−0.31 to 0.12)	.39	−0.09 (−0.42 to 0.24)	.61

^a^SE: standard error.

^b^Subgroup defined by having weight loss pledge 1 and 2.

^c^Multilevel mixed effect linier regression with municipality as a random effect.

^d^Participants with pledge regarding smoking habits was left out.

## Discussion

### Principal Findings

The primary aim of the study was to evaluate whether using a Web- and app-based tool for 38 weeks could stimulate beneficial changes on body weight and other anthropometric markers. We found that the SoSu-life group had lost −1.01 kg more body weight and −0.8% more body fat percentage than the control group at 38 weeks. The weight loss subgroup had an even larger decrease in body weight of −1.64 kg. These results correspond well with results from other studies [2,11,13,25]. These results suggest that a Web- and app-based weight loss tools can provide modest but statistically significant effects (−0.68 kg) compared with other weight loss methods (face-to-face-contact not included) [12]. The lower than expected completion rate may, however, have led to a lower statistical power than originally estimated.

### Interpretation

Overall, these results seem to correspond well with results from other workplace interventions studies [14]. The results indicate that the first 16 weeks of using the tool were the most effective. This is very much in line with other studies that have shown more favorable results in shorter periods with a Web-based weight loss tool [26,27]. In this study, some of the effects may be due to the competitions, with prizes, and the emphasis on developing healthy habits together with colleagues in the first intervention period. The lack of social support associated with Web-based tools has previously been reported as a problem [28]. The SoSu-life tool distinguishes itself from most other Web-based tools by having incorporating social support from known colleagues, both at the workplace and in a Web-based forum, and our findings indicate the importance of social features when addressing this target group.

We found that some of the weight loss and decrease in body fat percentage was maintained throughout the 38 weeks. However, it is debatable whether a period of 38 weeks is long enough to determine the long-term effects of a Web- and app-based weight control tool. A meta-analysis by Kodama et al [12] suggests that the study period must exceed 12 months if it is to determine long-term effects. Only a few studies in this meta-analysis exceeded 12 months and these studies did not find any positive effects in using the Web-based tools [12]. The SoSu-life study shows that it is possible to maintain a weight loss for a relatively long period with the use of a Web- and app-based tool, but it cannot predict longer term effects.

The degree of motivation of participants is likely to influence results. Most previous comparable studies have recruited voluntary participants who are overweight or obese, and who have the specific intention to lose weight [25,26,29]. In this regard, our study differed significantly from previous studies. We included as many participants as possible at each workplace, regardless of their initial health status and motivation. We have therefore recruited participants with varying degrees of need for behavioral change, and different levels of motivation to change behavior. We therefore speculate that some participants took part in the study simply because their colleagues signed up and out of curiosity alone, and not necessarily with a wish to change lifestyle, and this may explain the small effects we observed.

There were fewer shift workers in the study population compared with the total population, and more shift and evening workers dropped out of the study than those working steady daytime or weekend schedules. This could be explained by the design of the intervention study, where the health examinations primarily took place during the day time, but the social elements in the tool and the group support from colleagues probably also worked better for those with a natural interaction with several colleagues during working hours.

We did not find any effects on blood pressure and cholesterol. We ascribe this to the rather high variation in these measures, as well as the modest effects that were shown in the anthropometric markers. The intervention needs to be further improved or applied for longer time periods if it is to induce more significant clinical benefits. Furthermore, other clinical outcomes, such as insulin sensitivity, should be investigated, and subgroup assessments might identify risk profiles that would benefit more than the average user.

The extent to which the study participants were capable of using the Web- and app-based digital tools was not considered in the study, so essentially all employees were considered eligible. The participants were only given a brief introduction as to how to use the tool. This particular group of people, with lower socioeconomic status and working day-to-day work with elderly residents, may well be less capable of using digital tools than the average population. Several participants had never used a mobile phone before and were not very comfortable with using a computer. This invariably created a nonuser group of participants in the intervention group. The results may thus not by fully ascribable to the actual use of the SoSu-life tool but also in part to increased focus on health in general. Furthermore, randomization was not blinded, and as both intervention and control group underwent three health examinations, participants in the control group may have received an incentive to make lifestyle changes by virtue of the information from their health examination.

The intervention was designed to be applicable to the social and health care workers’ general workplace. However, if implemented in a practical context, the health examinations might very possibly be curtailed, or dropped, due to the extra cost, and the introduction to the tool would probably take place on the Web, so the effects of the tool would probably be smaller. The SoSu-life tool was designed to run for 38 weeks. For the intervention to be sustainable in the long term, it would probably require additional resources from the developers, sponsors, and the workplaces. New content would be needed to be added to the tool, repeated team competitions with accompanying prizes (from the sponsor) would probably have to take place, and management would have to make an effort to motivate employees to keep participating and using the tool. Regular health checks for the employees could possibly be conducted by managers or other staffs, as most of them are trained health professionals.

### Conclusions

In conclusion, the SoSu-life Web- and app-based intervention in employees in the social welfare and health care sector resulted in a lowering in body weight, body fat percentage, and waist circumference, particularly among those that were specifically motivated for weight loss, and during the first 16 weeks of intervention, when encouraged with group-based prizes in a competition. The longer term effects at week 38 were modest and associated with a relatively high dropout. To maintain the effects beyond an initial period will probably require further reinforcement of the intervention.

## Appendix

Multimedia Appendix 1 Supplementary material.

Multimedia Appendix 2 Presentation of the SoSu-life project and tool given to the participants in the intervention group at the information meeting. NB In Danish.

Multimedia Appendix 3 The CONSORT CONSORT eHealth checklist (v. 1.6.1) for the study.

## Abbreviations

ITT: intention-to-treat
SD: standard deviation
WHO: World Health Organization
