# Supplementary material for: Effect of a Nine-Month Web- and App-Based Workplace Intervention to Promote Healthy Lifestyle and Weight Loss for Employees in the Social Welfare and Health Care Sector: A Randomized Controlled Trial
Source: J Med Internet Res. 2017 Apr 10;19(4):e108. doi: 10.2196/jmir.6196 (PMC5404146; doi:10.2196/jmir.6196)
Supplement: Multimedia Appendix 1 [file jmir_v19i4e108_app1.pdf]

Supplementary material

Detailed description of sampling and study design.

The study was conducted in six municipalities in Denmark (Frederiksberg, Halsnæs, Hillerød, Hvidovre, Ishøj and Sønderborg). In each municipality two to four nursing homes were randomized to either SoSu-life or control. Those in SoSu-life group were given access to the SoSu-life tool for 38 weeks, whereas those in the control group did not receive any help. Those municipalities with an even number of nursing homes were randomized 1:1 and municipalities with an odd number of nursing homes randomized 2:1. In total 20 nursing homes agreed to participate in the study, and 12 nursing homes or home care units ended up in the intervention group and 8 in the control group. The study was divided into 2 distinct intervention periods. An initial 16 week period including a competition among the participating nursing homes, and a subsequent 22 week period without a competition. The enrolment of nursing homes was done consecutively, so that the baseline examination on the first participants was done in August 2011, whereas the last 38 week examinations were done around July 2012. If the participants did not own a smart phone, they could lease one free of charge from the project. Due to tax regulations on multi-media items, the leased phone containing the SoSu-life app could only be used for the SoSu-life project and not for personal use, such as making calls and sending text messages. Generally, the employees allowed the participants to use the SoSu-life program during work hours throughout the project period, and the participants were encouraged to use it at home, and during work free hours as well. The control groups were given access to the SoSu-life after the 38 week intervention period.

Recording of anthropometric measures and blood pressure

All measurements were performed by project staff. Weight was measured to the nearest 0.1 kg on a digital scale (TANITA, Electronic scale WB – 100MA/WB – 110MA III). Standing height was measured in cm to the nearest 0.5 cm with the participant positioned against the wall (TANITA, Invicta Plastics Limited, Oadby, Leicester, LE2 4LB, England). Bioimpedance was used to estimate total body fat percentage (OMRON, Body Fat Monitor, BF306). The device could measure a total of 49.9 % in body fat percentage, at the maxumum. Waist circumference was measured horizontally

at the navel to the nearest 0.5 mm with measuring tape. Further, hip circumference was measured horizontally at the widest point between the hip and the buttocks to the nearest 0.5 cm with a measuring tape. Blood pressure was measured with a digital blood pressure device (KIWEX, Automatic Blood Pressure Monitor, Model UA-787 Plus, A&D Medical Japan, Tokyo). Throughout all the measurements, the participants were barefooted, if possible, and wore underwear. Participants were encouraged to fast approximately three hours before the examination, although consumption of water, coffee, or tea was allowed. Time for the last meal was noted if the last meal or drink was consumed less than 360 minutes prior to the examination.

Blood samples

Blood samples were collected using a finger prick test (BIO Microcrotainer, contact-activated Lancet). Glucose was measured with HemoCue Glucose 201 RT, and total cholesterol was measured with Accutrend Plus, COBAS GCTL-mmol/L. The glucose levels are not included in the analyses because the manufacturer informed us of a possible error in the micro-cuvettes used for the sampling and analyses glucose in the blood.

Details on the weight loss part of the SoSu-life tool

The weight loss tool is based on a unit system where a specific amount of food is equal to a specific amount of units, based on amounts of calories and nutritional composition. Based on the users' height and weight the daily energy level is calculated and a suggested amount of unit per day is being suggested for weight loss. The user register his or her diet intake and daily exercise and the program gives feedback on the energy balance of the day with a green code for proper energy balance and a red code for too high energy intake.

The SoSu-life tool food registration feature is designed to support healthy eating and/or a weight loss pledge. This feature, called the Unit System was developed by the University of Copenhagen. On the basis of body weight, gender, age, and the specified desired weight loss entered by the study participant while taking the pledge, the program calculates an estimated daily maximum energy intake. The system contains a full food database, where each item is

defined by energy units of 250 KJ/unit. An energy unit was added to the daily energy balance as food and drinks, but these units were also subtracted by the amount of physical activity reported. Furthermore, the units had a colour that specified the dominating macronutrient in the foodstuff. The program recommended a certain distribution of colours to obtain the healthiest and most saturating diet. This system allowed the participant to eat the food they prefer during weight loss. Physical activity was registered by registering time and type of activity, and the amount of energy expended was then calculated in units of 250 kJ.

Description of the SoSu-life intervention

The following description of the SoSu-life intervention is a description of the items according to the TIDieR checklist that have not already been described or not been fully described in the main paper. The intervention was developed in 2011 before a TIDieR checklist and guide was available (2014). Therefore it is not possible to answer all items fully but the following description is our best attempt.

1. Brief name

SoSu-life, a web and app-based workplace health promotion tool to help weight loss and well-being for social and health care workers.

2. Why. Rationale, theory, or goal of the elements essential to the intervention

By assisting the social and health care workers engage in a health promotion project where group interactions play a very important part they can support each other in creating healthy habit changes both during and outside of working hours. Furthermore, they are provided with a digital tool that can help assist the individual user to achieve weight loss, eat healthier, improve fitness, get a stronger body, quit smoking or decrease number of cigarettes.

A simple model of the idea behind the SoSu-life tool.

Simple model of intended mechanism in the SoSu-life-tool

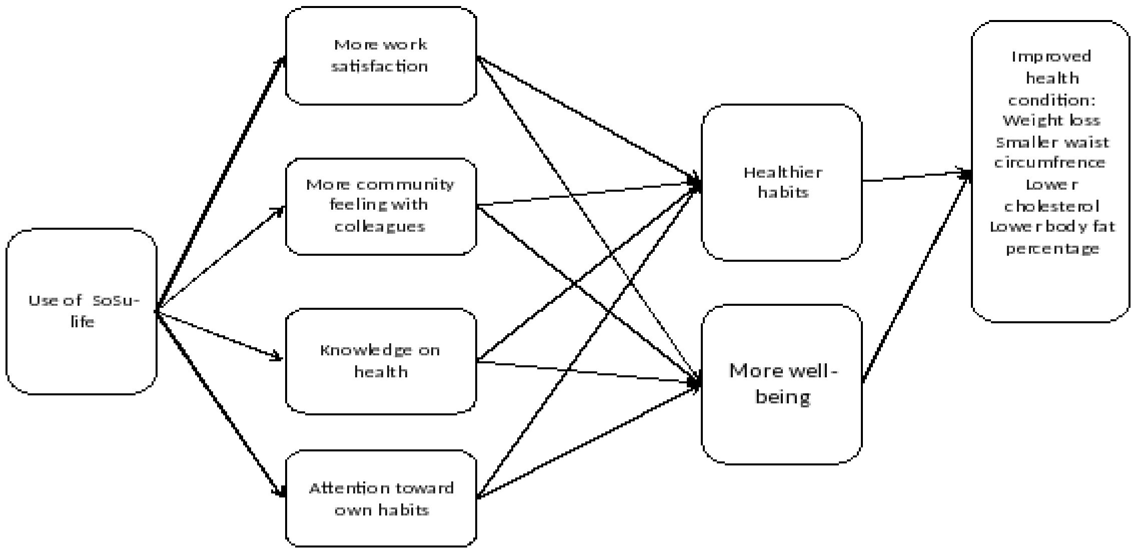

The different features and their purpose in the SoSu-life tool are listed below.

The social features

-Team competition: When the user solves the challenges given by the program and their colleagues, points are awarded to the team (work) in the competition against all other participating nursing homes. General use of the program is also rewarded with points. During the first 16 weeks of the intervention it was possible to win a prize once a month. After the first 16 weeks the nursing homes that collected the most points won the main prize.

- Weekly assignments: These are weekly tasks to be solved together with colleagues or individually. All participants must solve the same task.

- Colleague Challenges: These are challenges sent from colleague to colleague. The challenges the participants could receive was depending on their own personal pledge.

- Groups: Establishing groups to cultivate communities of interest with colleagues and other users.

- Forums: Free debate on all topics with all users of the program.

The individual features to help support the pledge

- Diet: Calorie calculator.

- Exercise: Calorie calculator.

- Smoking cessation: Tool to help cut down on cigarettes or quit smoking.

- Well-being: Tool to enter today's mood.

- My habits: Tool to get inspired to try out new habits.

- SMS help: Tool to receive personal SMS messages with reminders of new habits or other self-selected topics.

- Quick input key: Easy input of daily meals, consumption of fruits and vegetables, water consumption and daily exercise.

- My Status: Statistics on weight development, smoking patterns and well-being.

- Weight and cm: Tool to enter body weight and waist circumference.

Other features

- Quizzes

- Tests

- Food recipes: Extensive library of recipes including online videos with tutorials.

- Articles: Knowledge, tips and tricks regarding food, exercise, smoking and well-being.

3. What (materials): Describe any physical or informational materials used in the intervention, including those provided to participants or used in intervention delivery or in training of intervention providers.

The intervention group were given access to the SoSu-life website and app (and provided free of charge with a smart phone if the participant did not already own one. The smart phone was blocked, so that it could only access and use the SoSu-life APP). The features are described in item 2 and in the manuscript.

The participants were given a 10-15 minutes personal introduction to the SoSu-life tool from a project team member.

Participants were also provided with a paper pamphlet with a description of the main features of the web and app. Before signing an informed consent the participant were also given an information folder on the research project.

4. What (procedures): Describe each of the procedures, activities, and/or processes used in the intervention, including any enabling or support activities

The participants received a weekly text message with information on the weekly group assignment and two weekly text messages with tips and tricks depending on the participants' pledge. In the message program in the SoSu-life tool, the participants received information on how to be successful with the use of the tool and updates on how the intervention was progressing. Every month they received an email with a newsletter about who had won the monthly competition and the relevant score in the team competition.
Participant could send a message to counselors regarding use of the program or questions or help regarding how to achieve the goal in their pledge.
The participant could also call a hotline during working hours with questions regarding the tool.

5. Who provided: For each category of intervention provider (for example, psychologist, nursing assistant), describe their expertise, background and any specific training given

The health examinations were performed by personal from the research group. They were all trained in how to measure all the clinical measures according to standard operating procedures developed for the study. The counsellors answering text questions from the users had a dietitian, smoking cessation consultant or sport trainer background.

6. How: Describe the modes of delivery (such as face to face or by some other mechanism, such as internet or telephone) of the intervention and whether it was provided individually or in a group

See item 3 and 4.

7. Where: Describe the type(s) of location(s) where the intervention occurred, including any necessary infrastructure or relevant features

The study was conducted in six municipalities in Denmark from August 2011 - July 2012. In each municipality two to four nursing homes were randomized to either the SoSu-life tool (called SoSu-life group) or to a control group. The randomization of the participating nursing homes was conducted continually over time within each municipality. Those municipalities with an even number of nursing homes were randomized in a 1:1 ratio, and municipalities with an odd number of nursing homes in a 2:1 ratio. Twelve of a total of 20 nursing homes were randomized to SoSu-life, and 8 were randomized to control. The randomization was performed in a simple blinded way (simple paper draw) by two of the study investigators and/or in a collaboration between investigators and staff at an initial meeting with local staff in each municipality. Each draw was observed by independent witnesses to observe that it was performed in a fair and unbiased way. The study was divided into two distinct periods for the SoSu-life group: an initial 16 week period including a team competition, and a subsequent 22 week period without a competition. Participants chose a pledge for each period the (Figure 1).

Figure 1. Overview of the SoSu-life study

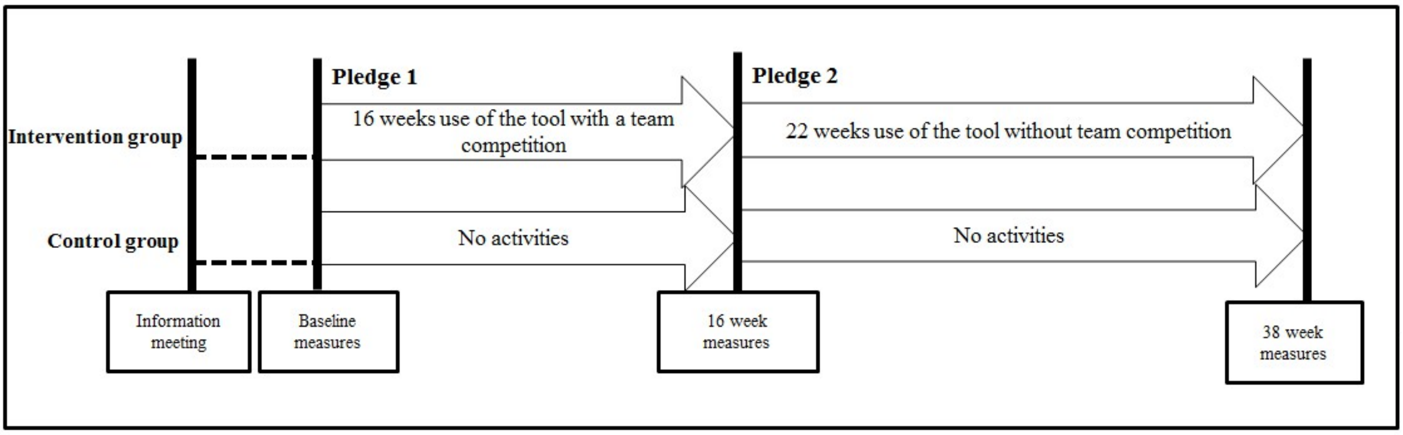

Participants were recruited August –September 2012 when an information meeting was held at each nursing home during work hours, after which each of the interested employees completed the informed consent form. Eligibility criteria for participation were that the employees had to work under conditions in accordance with a FOA-negotiated agreement.

8. When and how much: Describe the number of times the intervention was delivered and over what period of time including the number of sessions, their schedule, and their duration, intensity or dose

The participants had access to the tool for nine month. Also see item 4.

9. Tailoring: If the intervention was planned to be personalised, titrated or adapted, then describe what, why, when, and how

Specific features in the SoSu-life tool was personalized according to pledge: “Colleagues challenges”, received text messages, “my habits” and messages in the inbox,

10. Modifications: If the intervention was modified during the course of the study, describe the changes (what, why, when, and how)

There were no modifications of the intervention during the study period.

11. How well (planned): If intervention adherence or fidelity was assessed, describe how and by whom, and if any strategies were used to maintain or improve fidelity, describe them

The team competition with the monthly prizes and the main prize after 16 weeks was designed to keep participants motivated to use the SoSu-life tool. The monthly prizes were delivered to the wining nursing home by staff from the project team.

12: How well (actual): If intervention adherence or fidelity was assessed, describe the extent to which the intervention was delivered as planned

The SoSu-life tool worked as planned during the whole intervention period. 52 % of the participant who initially signed up for the study dropped out. The point system incorporated in the tool was an indication on whether or not the participants used the tool. Figure 1 shows how many point that on average were earned during the intervention period.

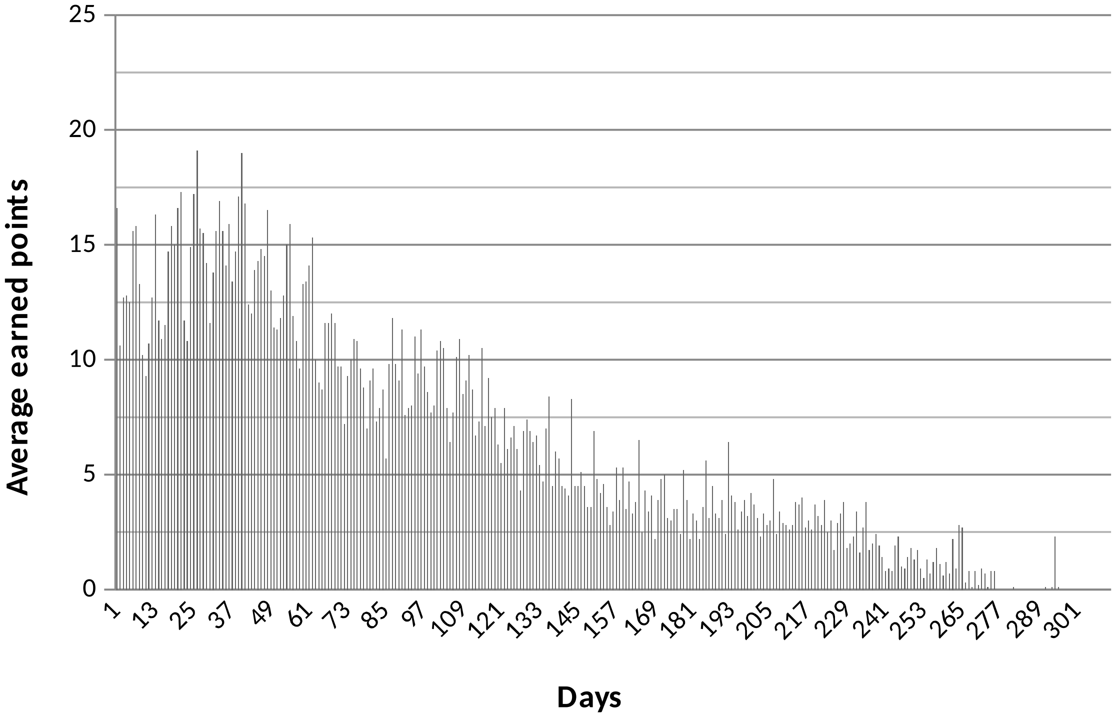

The figure shows that the use of the tool declined during the intervention period especially after the first 16 weeks (around day 112).

Comparative analysis of the completing participants vs. the drop out group

		Completing participants[a]	Drop out[a]	p-value
Age (in years)		46.9 ± 9.9	46.8 ± 10.6	0.944[b]
Females		250 (92.94 %)	272 (91.58 %)	0.884[c]
Work schedule[d]				
	Day duty	213 (79.5 %)	199 (68.2 %)	0.026[c]
	Evening duty	32 (11.9 %)	53 (18.2 %)	
	Night duty	11 (4.1 %)	13 (4.5 %)	
	Varying shifts	12 (4.5 %)	27 (9.3 %)	
Marital status[e]				
	Single	79 (29.5 %)	97 (33.65)	0.245[c]
	Cohabitate	189 (70.5 %)	192 (66.4 %)	
Children in the household[f]				
	Yes, 1 child	74 (27.7 %)	61 (20.9 %)	0.066
	Yes, 2 children	45 (16.9 %)	61 (20.9 %)	
	Yes, 3 or more	33 (12.4 %)	26 (8.9 %)	
	No	115 (43.1 %)	144 (49.3 %)	
Height (cm)		165.9 ± 7.3	165.7 ± 7.6	0.736
Body weight (kg)		73.8 ± 15.3	75.6 ± 16.7	0.183
Waist circumference (cm)[g]		92.2 ± 13.0	95.2 ± 13.5	0.007
Hip circumference (cm)		102.7 ± 10.8	103.8 ± 11.3	0.252
Total cholesterol (mmol/L)[h]		5.3 ± 0.9	5.4 ± 0.9	0.198
Body fat percentage (%)[i]		35.2 ± 7.4	36.02 ± 7.7	0.919
Systolic BP (mm Hg)		129.8 ± 16.5	130.9 ± 18.0	0.454
Diastolic BP (mm Hg)		81.5 ± 9.8	82.8 ± 11.4	0.137

[a]Completing participants, n=269, dropout, n=297 unless other is specified

[b]Data are compared between groups using Students *t*-test.

[c] Data are compared between groups using chi-square test

[d] Completing participants, n=268, dropout, n=292

[e] Completing participants, n=268, dropout, n=289

[f] Completing participants, n=267, dropout, n=289

[g] Completing participants, n=267, dropout, n=297

[h] Completing participants, n=263, dropout, n=297

[i]Body fat percentage device had a maximum measurement range of 49.9 %, Completing participants, n=263, dropout, n=297

ITT results

Intention to treat baseline carried forward results

	Changes from week 0 to week 16 and from week 0 to week 38	n	SoSu-life Mean ± (SE)	n	Sub-group with weight loss pledge[c] Mean ± (SE)	n	Control Mean ± (SE)	Adjusted difference[b] btwn SoSu-life group and control (95% CI)	*P*-value[b] btwn SoSu-life group and control	Adjusted difference, btwn sub-group[c] and control (95% CI)	*P*-value[b] btwn sub-group[c] and control
Body weight [a] (kg)											
	week 0 to 16	304	-0.96 ± 0.14	149	-1.19 ± 0.22	211	0.06 ± 0.13	-1.01 (-1.40, -0.62)	<.001	-2.36 (-1.40, -0.62)	<.001
	week 0-38	304	-0.48± 0.15	149	-0.56 ± 0.23	211	0.08 ± 0.33	-0.56 (-1.05, -0.08)	.02	-0.64 (-1.24, -.04)	.04
Body fat percentage[a]											
	week 0-16	296	-0.43 ± 0.11	143	-0.34 ± 0.14	206	0.01 ± 0.10	-0.48 (-0.79, -0.17)	.003	-0.35 (-0.67, -0.02)	.036
	week 0-38	296	-0.64 ± 0.15	143	-0.42 ± 0.13	206	-0.35 ± 0.14	-0.28 (-0.71, 0.15)	.20	-0.07 (-0.46, 0.32)	.72
Waist circumference[a] (cm)											
	week 0-16	304	-1.16 ± 0.24	149	-1.43± 0.39	210	-0.51 ± 0.26	-0.77 (-1.48, -0.06)	.03	-0.97 (-1.85, -0.09)	.03
	week 0-38	304	-0.40 ± 0.23	149	-0.42± 0.35	210	-0.10 ± 0.29	-1.01 (-1.72, -0.31)	.005	-0.86 (-1.72, -0.01)	.053
Systolic blood pressure (mm HG)											
	week 0-16	355	-1.89± 0.64	154	-0.88± 0.77	211	-1.69± 0.49	0.20 (-1.39, 1.78)	.81	1.01 (-0.94, 2.96)	.31
	week 0-38	355	-1.64± 0.50	154	-1.51± 0.73	211	-3.30± 0.77	1.31 (-0.43, 3.04)	.14	1.61 (-0.54, 3.76)	.14
Diastolic blood pressure (mm HG)											
	week 0-16	355	-1.53 ± 0.35	154	-1.01 ± 0.55	211	-1.16 ± 0.42	-0.48 (-1.58, 0.63)	.38	0.06 (-1.28, 1.41)	.93
	week 0-38	355	-1.19 ± 0.31	154	-1.18 ± 0.52	211	-1.44 ± 0.40	0.14 (-0.86, 1.13)	.79	0.26 (-1.00, 1.52)	.69
Total cholesterol (mmol/L)											
	week 0-16	347	0.04 ± 0.04	148	0.05 ± 0.05	204	0.14 ± 0.05	-0.09 (-0.22, 0.03)	.52	-0.09 (-0.24, 0.06)	.26
	week 0-38	347	0.25 ± 0.31	148	-0.07± 0.05	204	-0.00 ± 0.05	0.27 (-0.54, 1.08)	.39	-0.07 (-0.20, 0.07)	.32

[a]Participants with pledge regarding smoking habits was left out.
[b]Multi-level mixed effect linier regression with municipality as a random effect
[c]Sub-group defined by having weight loss pledge 1 and 2.
