# Supplementary material for: Effect of a Nine-Month Web- and App-Based Workplace Intervention to Promote Healthy Lifestyle and Weight Loss for Employees in the Social Welfare and Health Care Sector: A Randomized Controlled Trial
Source: J Med Internet Res. 2017 Apr 10;19(4):e108. doi: 10.2196/jmir.6196 (PMC5404146; doi:10.2196/jmir.6196)
Supplement: Multimedia Appendix 2 [file jmir_v19i4e108_app2.pdf]

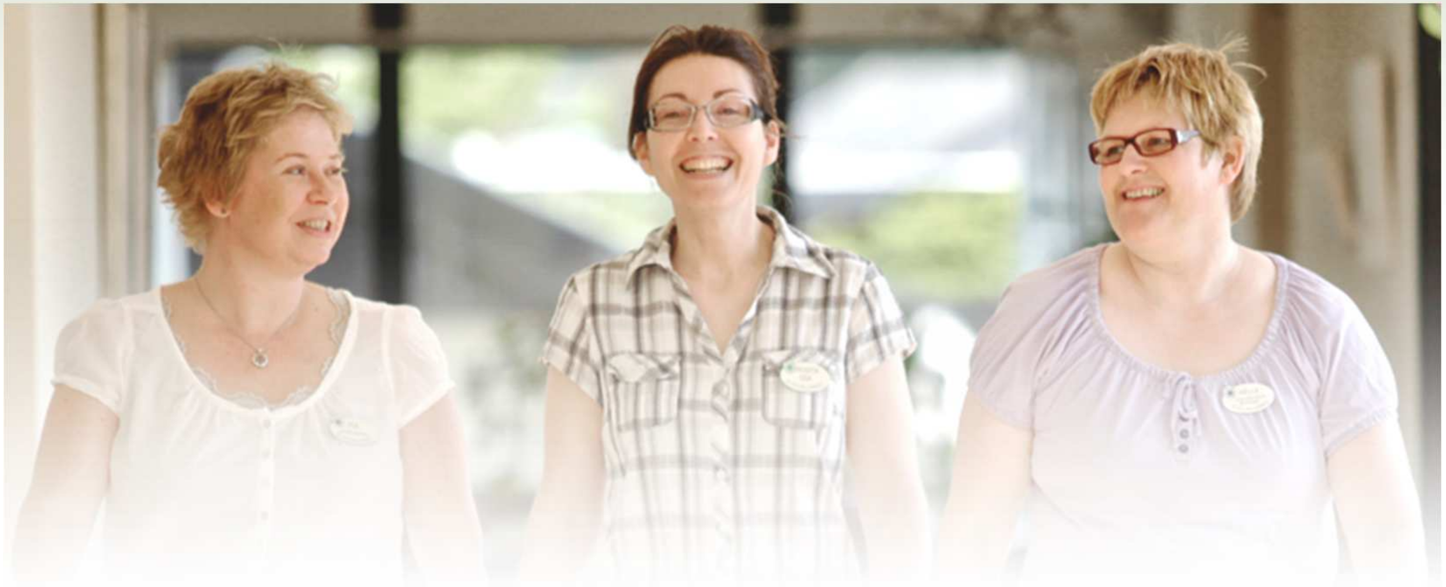

En ny vej til
større overskud, trivsel og velvære

Mandag den 24. september

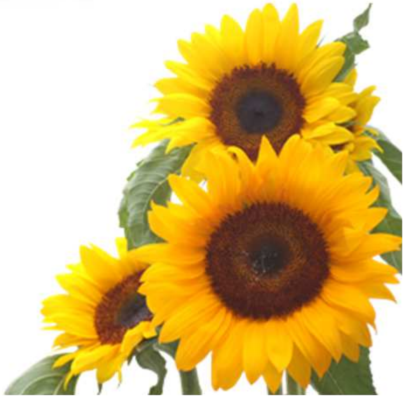

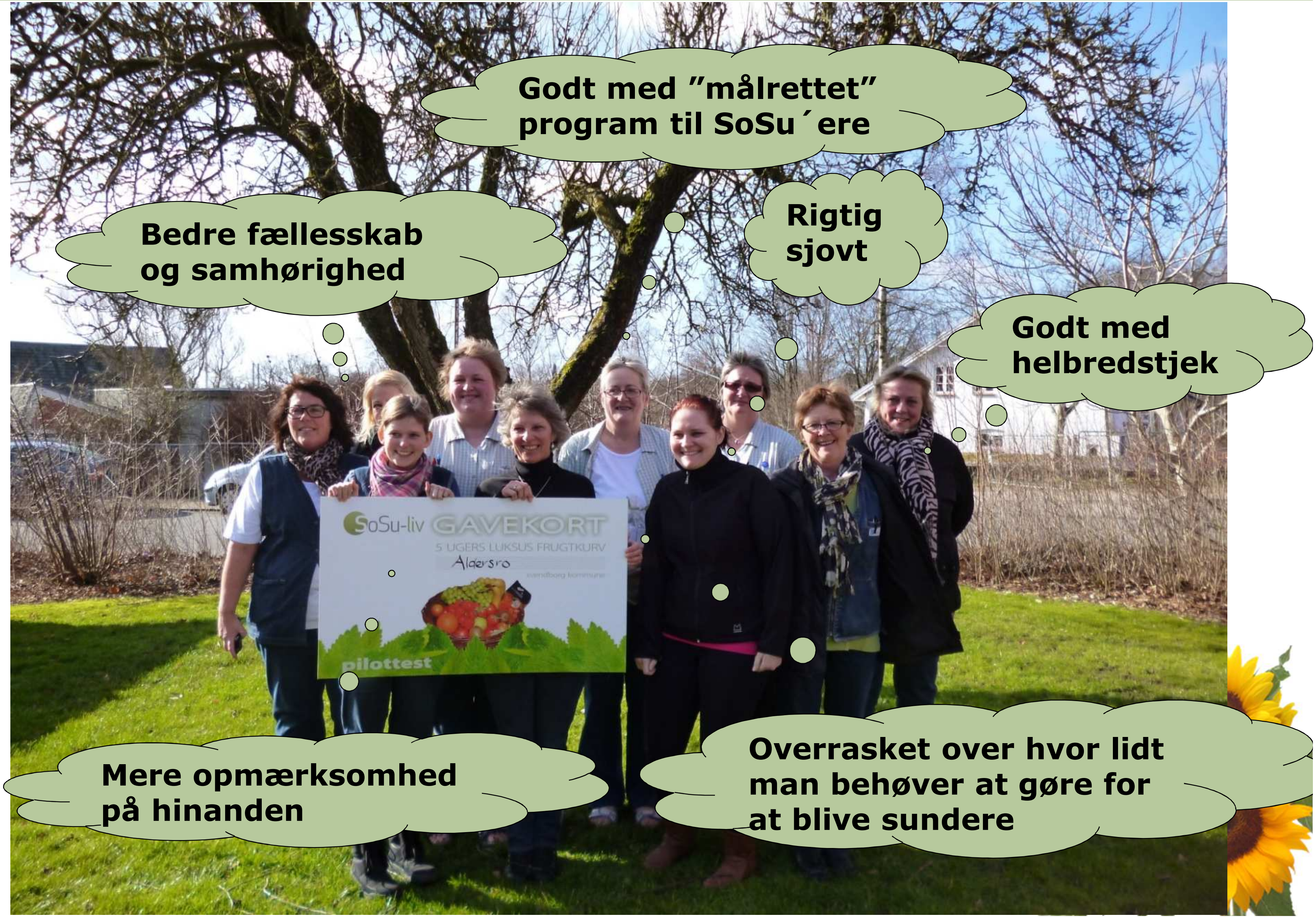
Godt med "målrettet" program til SoSu´ere
Rigtig sjovt
Bedre fællesskab og samhørighed
Godt med helbredstjek
SoSu-liv GAVEKORT
5 UGERS LUKSUS FRUGTKURV
Aldersro
pilottest
Mere opmærksomhed på hinanden
Overrasket over hvor lidt man behøver at gøre for at blive sundere

Hvad er SoSu-liv ?

- Et gratis program til mobiltelefon og computer:
  - om kost, motion, rygning og velvære
- SoSu-liv kan hjælpe til at fremme jeres
  - trivsel og fællesskab
  - helbred og velvære

Vi ønsker, at alle offentligt ansatte på en FOA-overenskomst kan få programmet som en del af deres arbejdsmarkedspension

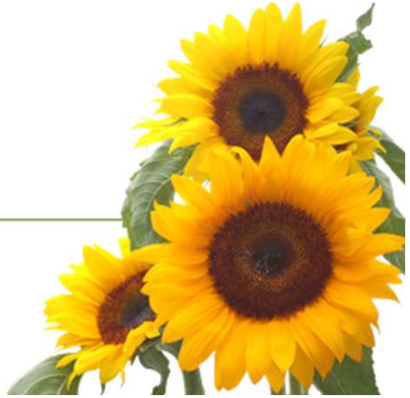

SoSu-liv

Programmet hedder SoSu-liv
Fungerer på hjemmeside og et program på en mobiltelefon

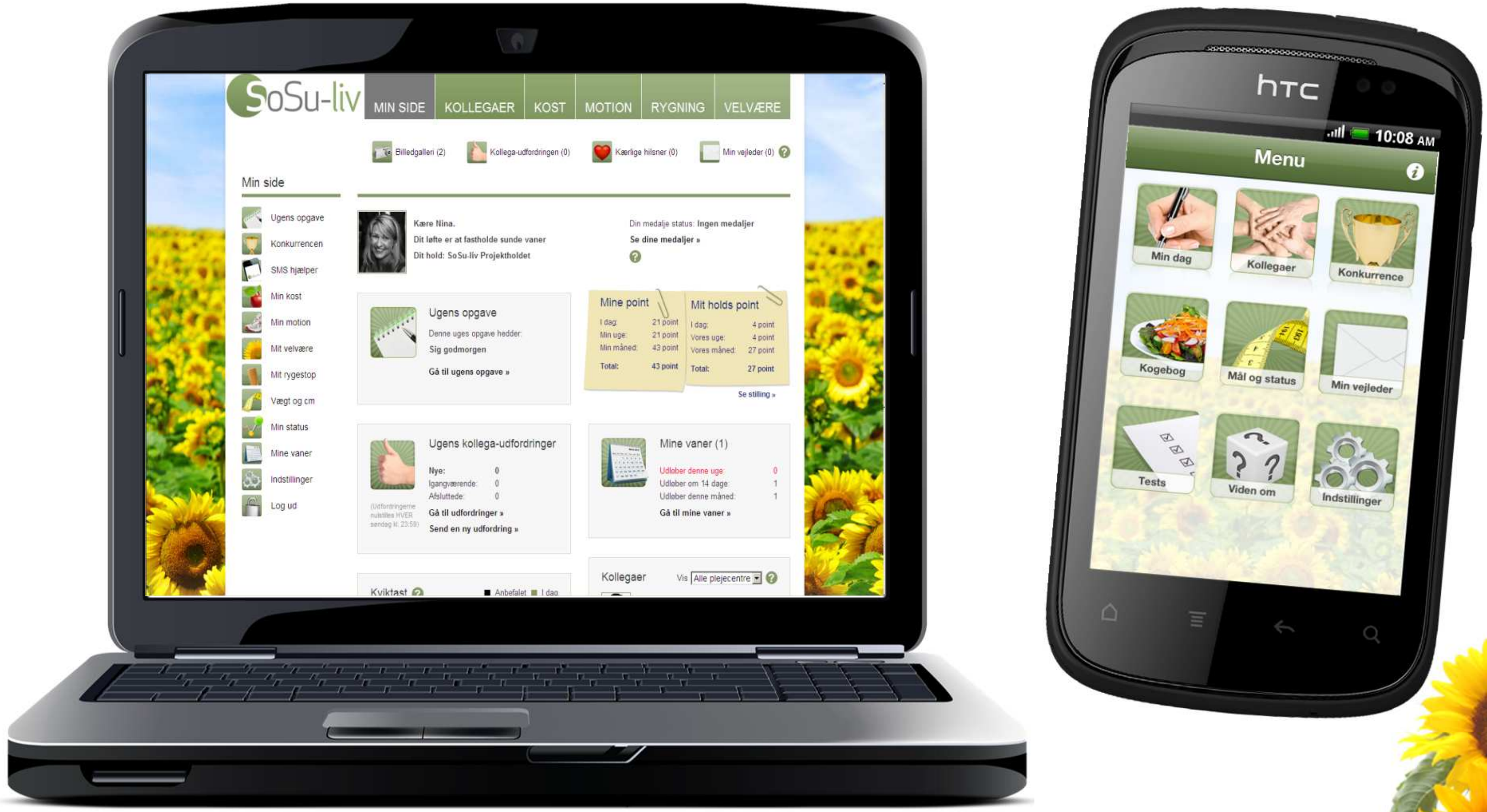

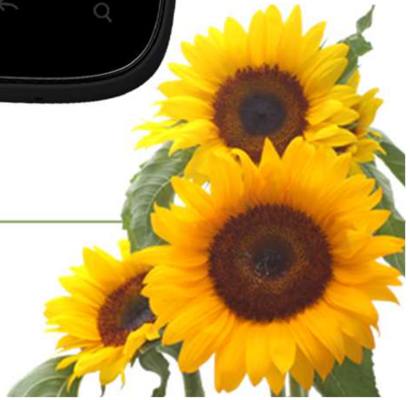

SoSu-liv på mobiltelefon

- Du får SoSu-liv på:
  - Arbejds smartphone
  - Private smartphone

- **Eller** du låner GRATIS en mobiltelefon

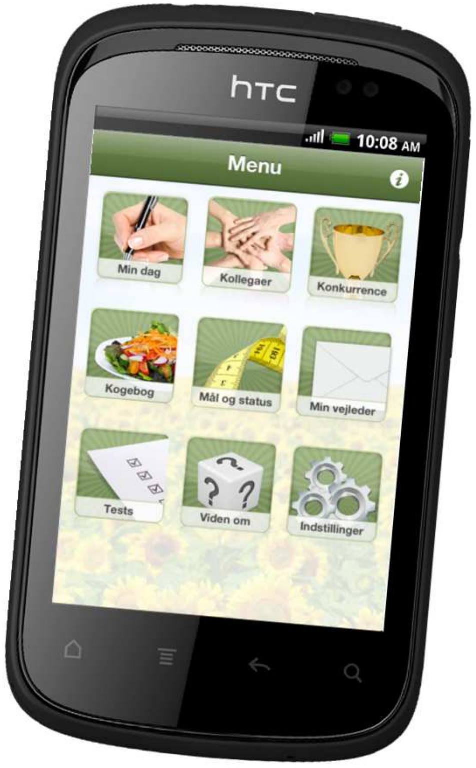

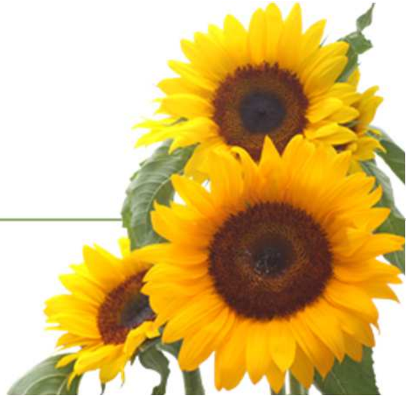

SoSu-liv

Hvad er nyt ved SoSu-liv programmet?

- Trivsel og sundhed er noget vi **gør sammen**
- Vi kan **hjælpe hinanden** med at blive **gladere, sundere** og **få mere overskud**

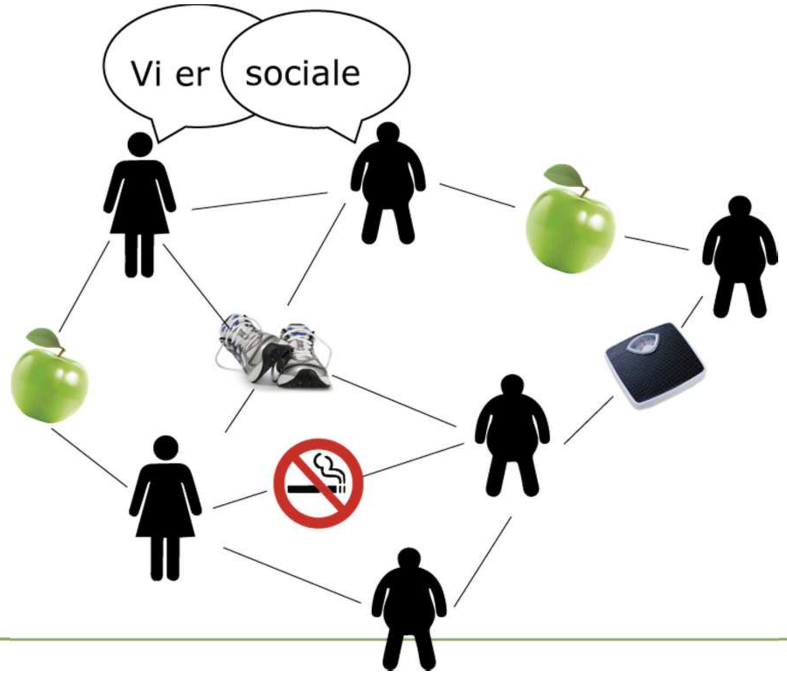

SoSu-liv

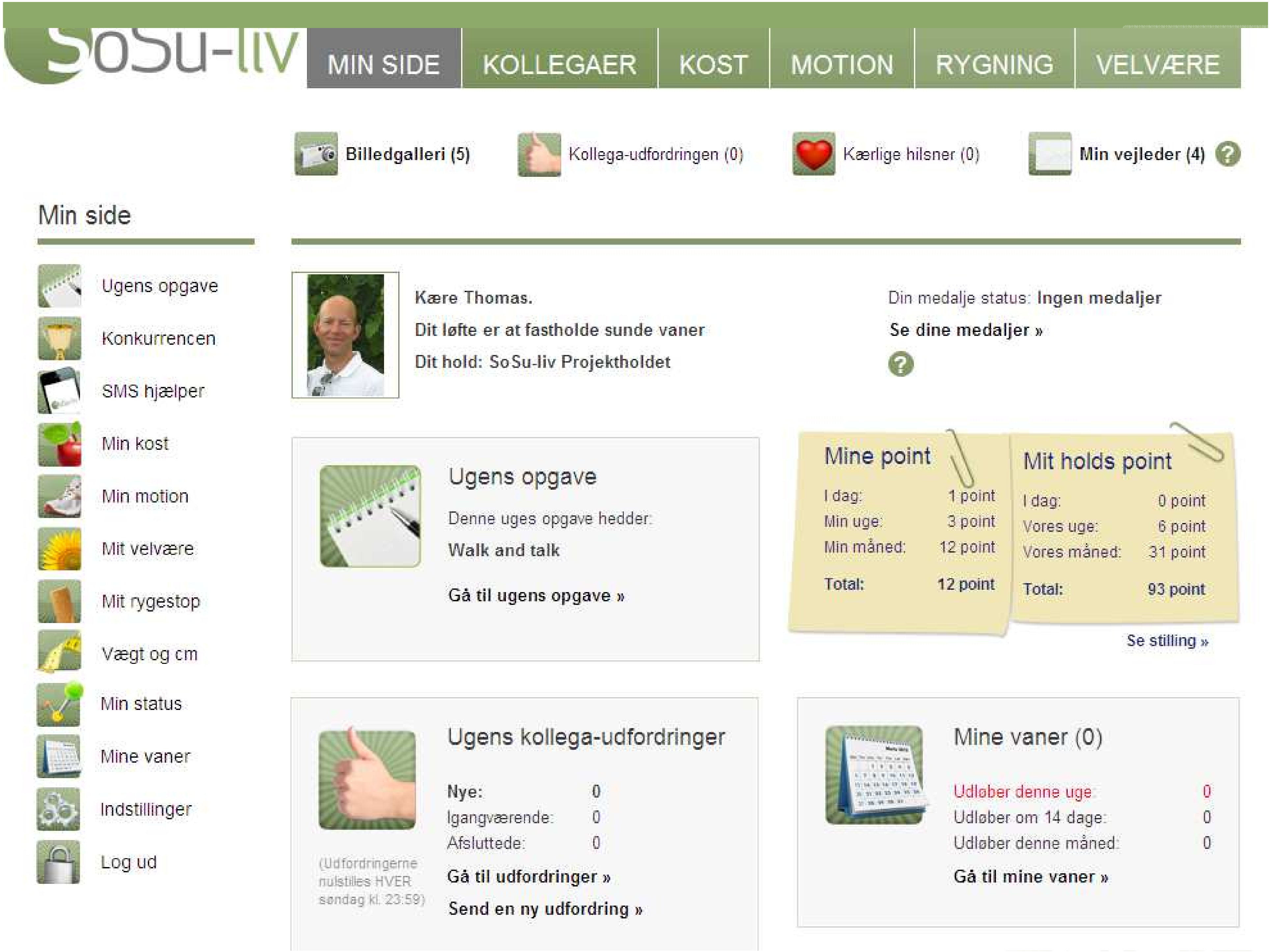
SoSu-liv
MIN SIDE
KOLLEGAER
KOST
MOTION
RYGNING
VELVÆRE
Billedgalleri (5)
Kollega-udfordringen (0)
Kærlige hilsner (0)
Min vejleder (4)
Min side
Ugens opgave
Konkurrencen
SMS hjælper
Min kost
Min motion
Mit velvære
Mit rygestop
Vægt og cm
Min status
Mine vaner
Indstillinger
Log ud
Kære Thomas.
Dit løfte er at fastholde sunde vaner
Dit hold: SoSu-liv Projektholdet
Din medalje status: Ingen medaljer
Se dine medaljer »
Ugens opgave
Denne uges opgave hedder:
Walk and talk
Gå til ugens opgave »
Mine point
I dag: 1 point
Min uge: 3 point
Min måned: 12 point
Total: 12 point
Mit holds point
I dag: 0 point
Vores uge: 6 point
Vores måned: 31 point
Total: 93 point
Se stilling »
Ugens kollega-udfordringer
Nye: 0
Igangværende: 0
Afsluttede: 0
Gå til udfordringer »
Send en ny udfordring »
(Udfordringerne nulstilles HVER søndag kl. 23:59)
Mine vaner (0)
Udløber denne uge 0
Udløber om 14 dage: 0
Udløber denne måned: 0
Gå til mine vaner »

Ugeopgaver

- I 16 uger er der ugeopgaver
- Ugeopgaver er øvelser inden for kost, motion eller velvære
- Øvelserne kan løses på ca. 5 minutter
- Alle på holdet laver den samme opgave

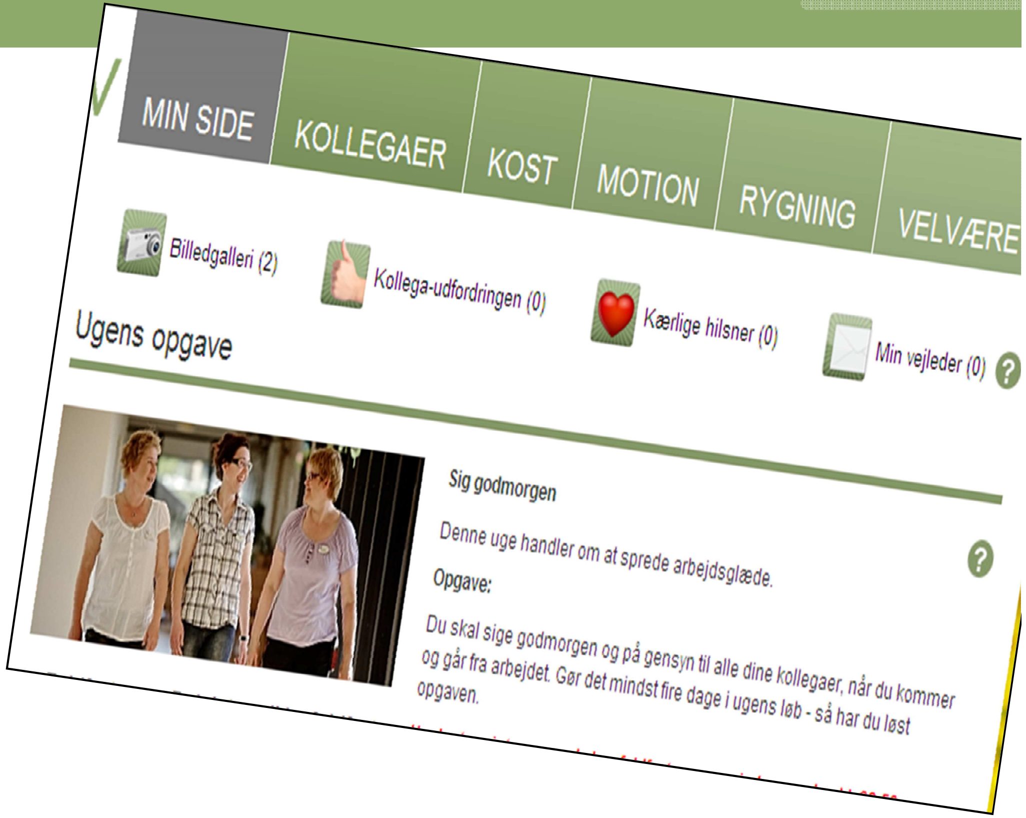

Opgaven åbnes mandag morgen kl. 00.01

Opgaven lukkes søndag aften kl. 23.59

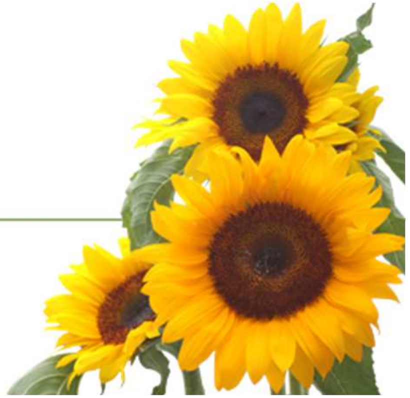

Ugens opgave: Sådan gør du ….

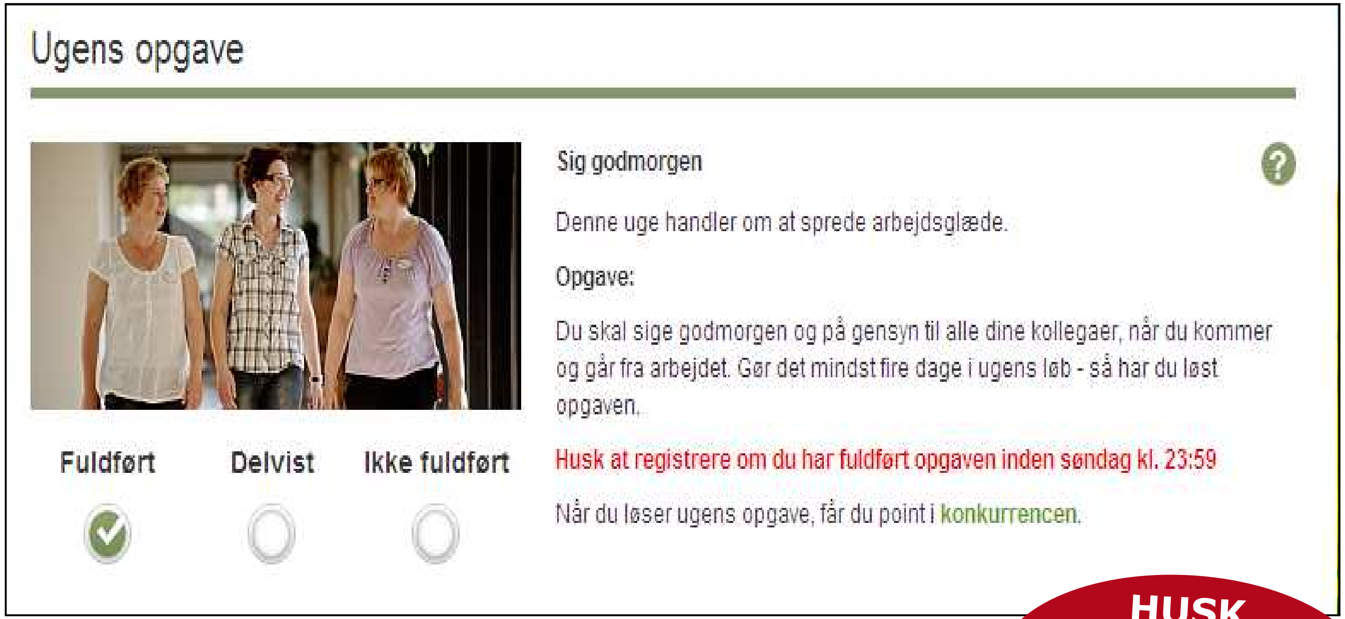

HUSK
at registrere,
at du har løst
opgaven

SoSu-liv

Kollegaudfordring

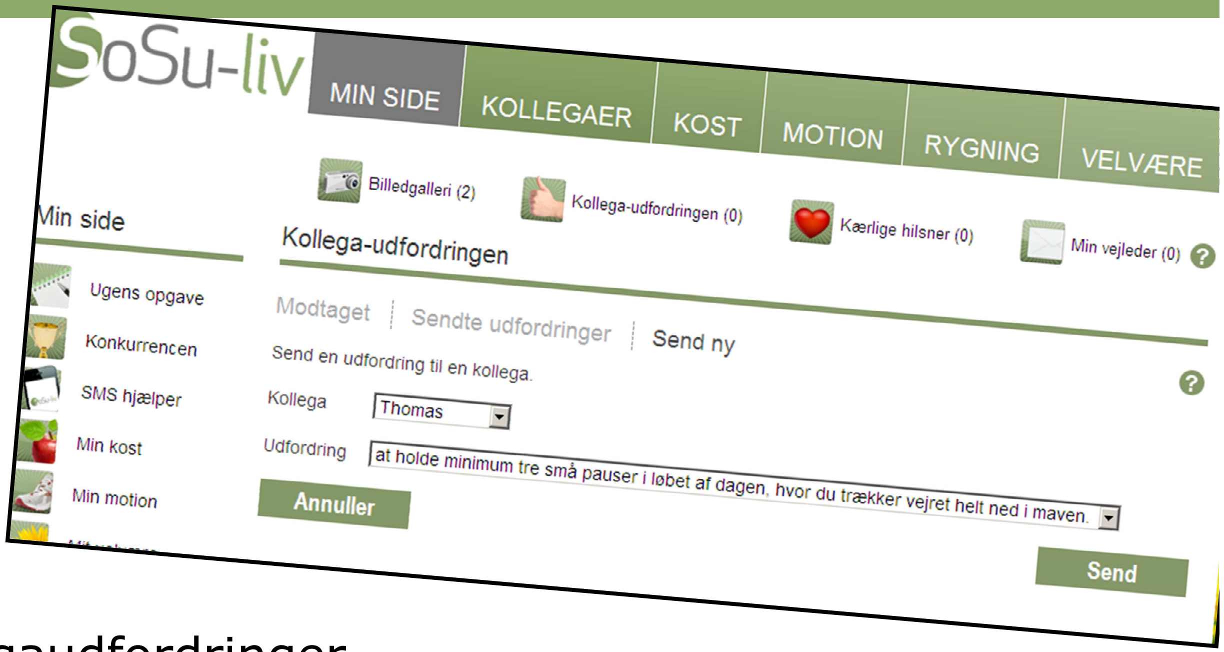

- I 16 uger er der kollegaudfordringer
- Du kan vælge imellem inden for: kost, motion, rygning eller velvære

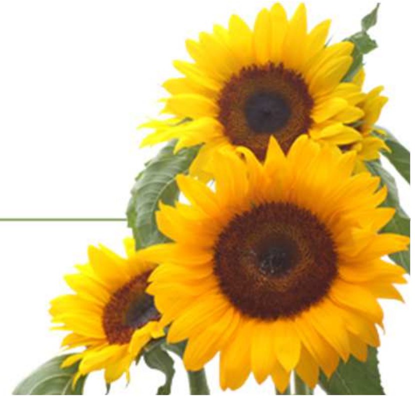

SoSu-liv

Kollegaudfordring: Sådan gør du ….

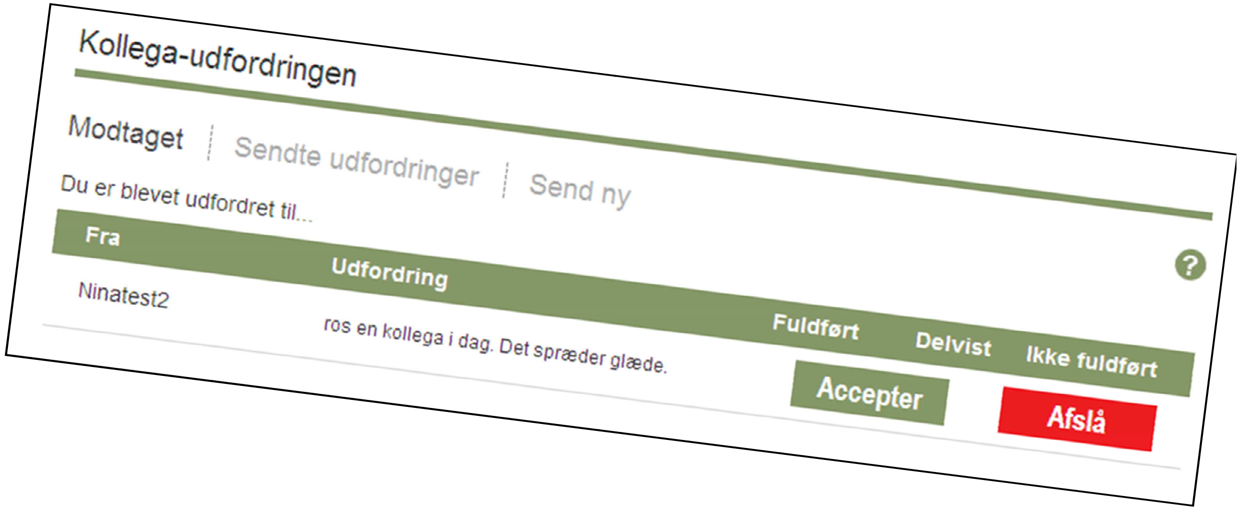

- 1. Du finder en kollega, som du vil udfordre
  - Du vælger selve udfordringen og sender den
  - Kollegaen skal nu acceptere din udfordring

2. Du skal også selv acceptere eller afslå dine egne udfordringer

HUSK at følge op på dine kollega udfordringer

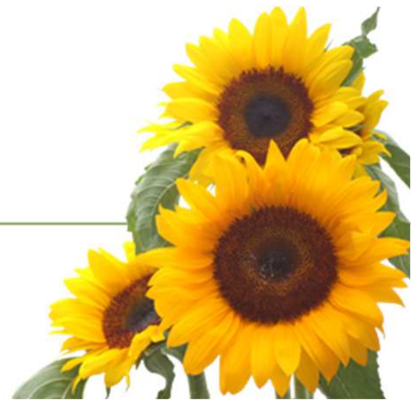

SoSu-liv

Vær opmærksom på dine kollegaer

- Du kan hjælpe – og blive hjulpet og underholdt

Send beskeder, opskrifter, artikler, quiz m.v. til alle deltagere i SoSu-liv

- Du kan motivere og opmuntre kollegaer

  - Grupper
  - Debatfora
  - Tage billeder
  - Kærlige hilsner

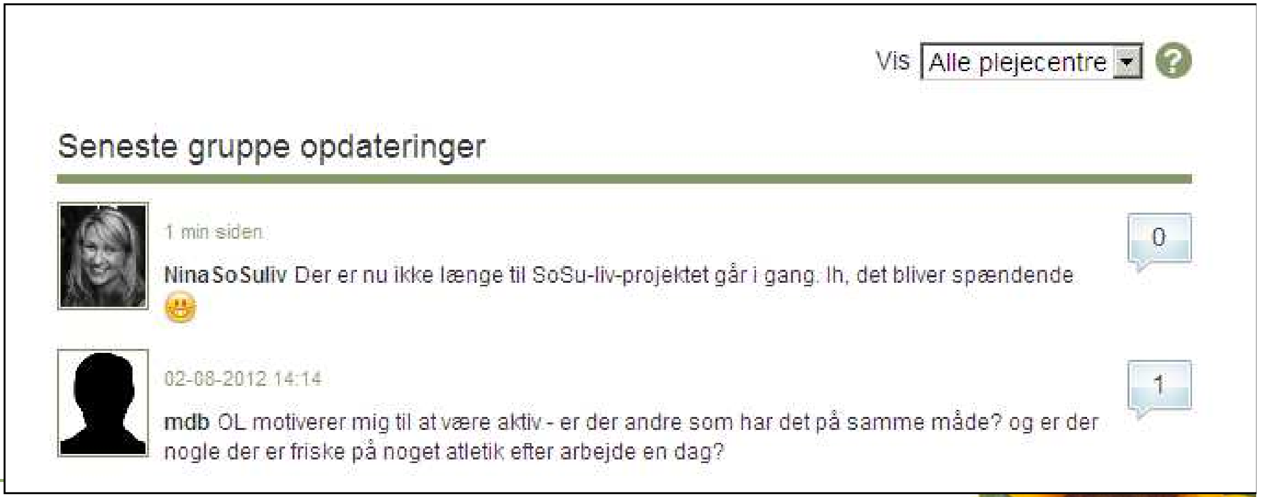

SoSu-liv

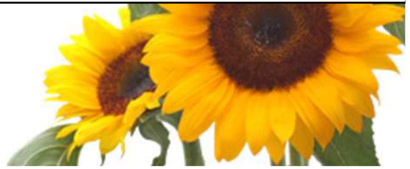

Husk dig selv…

- Du kan finde inspiration til nye vaner under ”Mine vaner”
- Du kan vælge nye vaner inden for kost, motion, rygning og velvære
- Du bestemmer selv, hvor længe du vil afprøve din nye vane

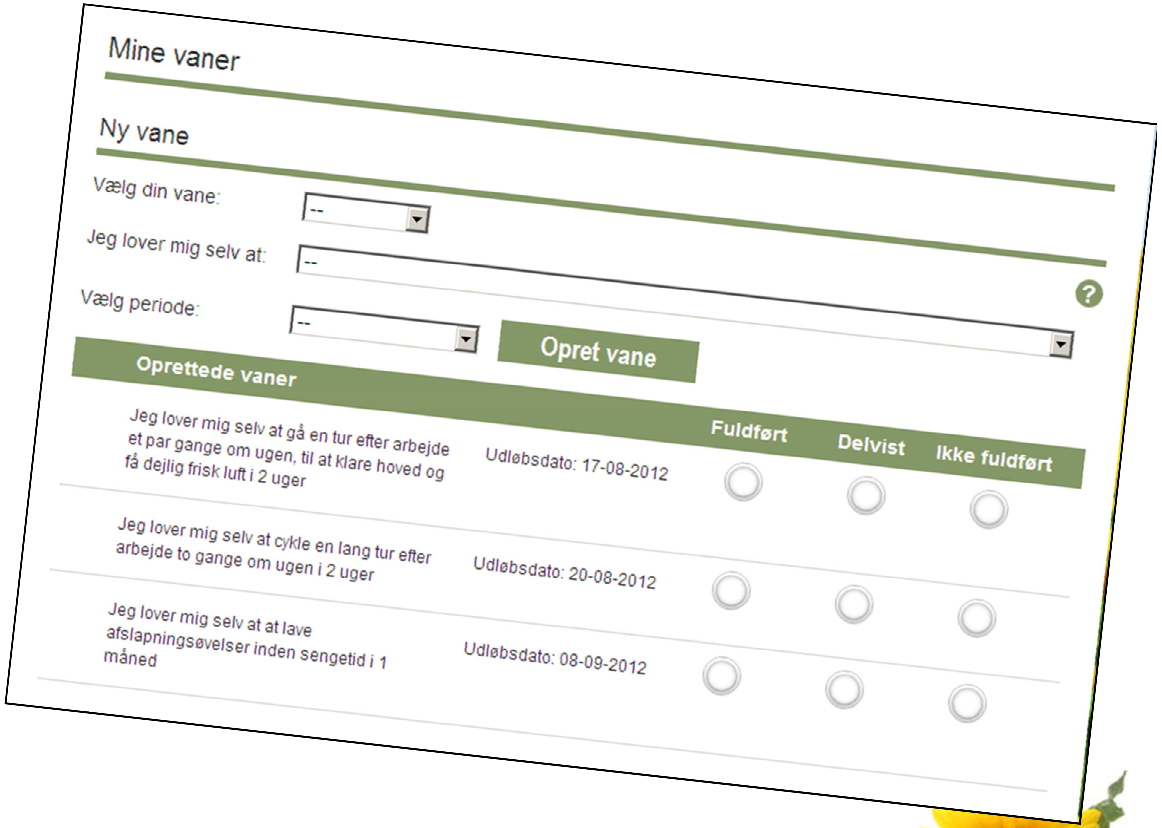

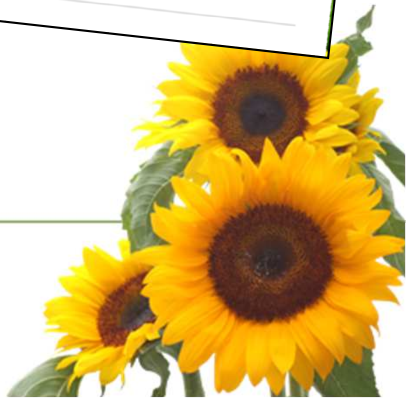

Sådan får du sms-hjælp

- Du kan oprette en SMS-hjælper til minde dig om de nye gode vaner

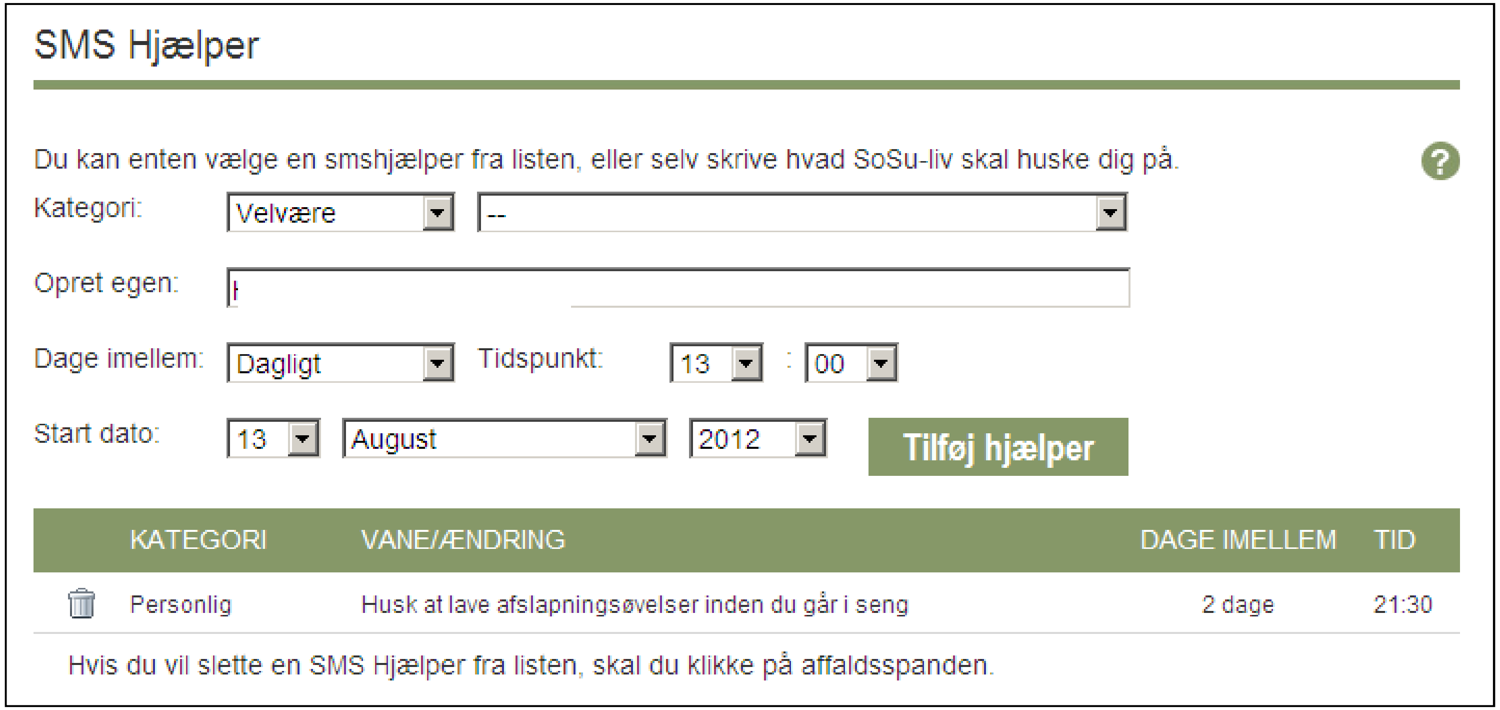

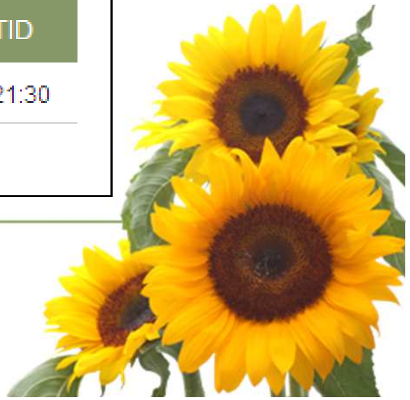

SoSu-liv

Sådan får du personlig hjælp

- Du kan få hjælp med dine spørgsmål:
  - Brevkassevejleder – en personlig coach

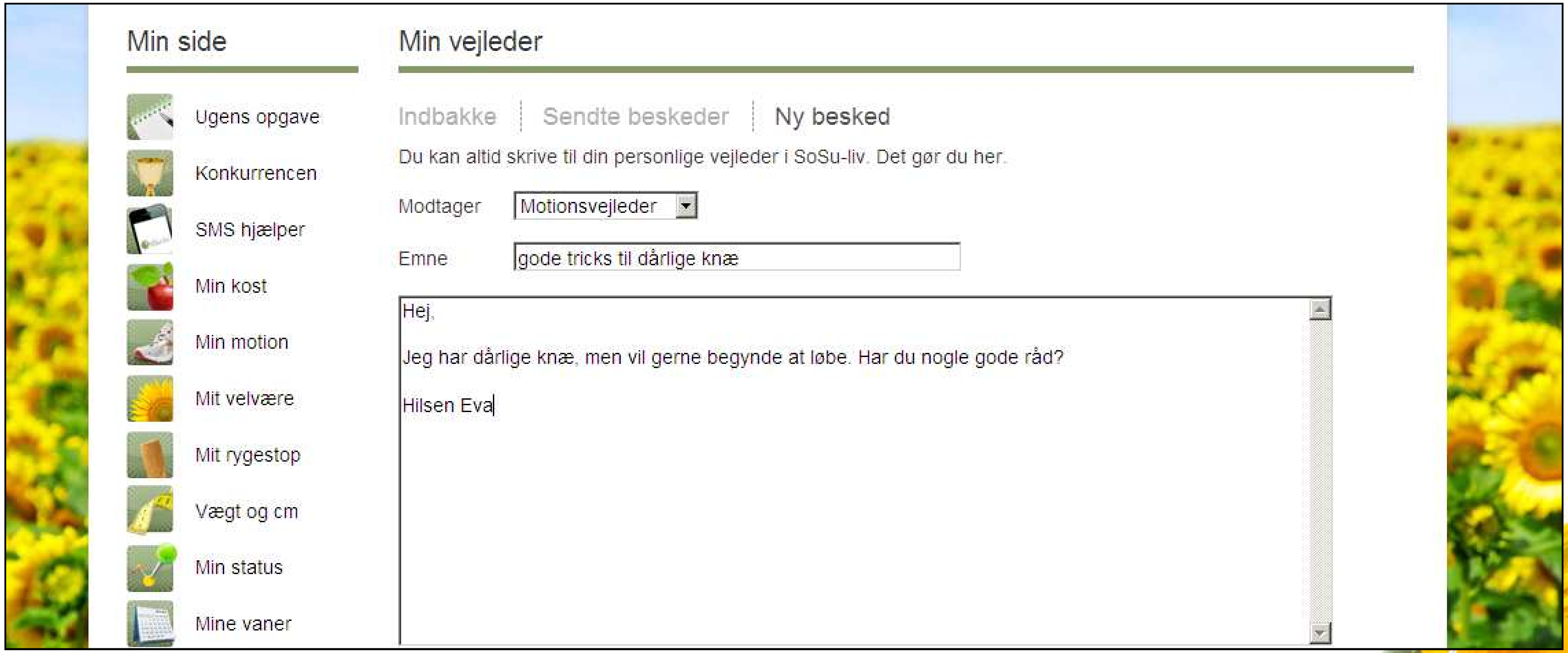

SoSu-liv

MIN SIDE | KOLLEGAER | KOST | MOTION | RYGNING | VELVÆRE

Min side > Min kost > **Dagens kost**

Billedgalleri (5) Kollega-udfordringen (0) Kærlige hilsner (0) **Min vejleder (4)** ?

Min side

- Ugens opgave
- Konkurrencen
- SMS hjælper
- Min kost
  - Overblik
  - **Dagens kost**
  - Madplan
- Min motion
- Mit velvære
- Mit rygestop
- Vægt og cm
- Min status
- Mine vaner
- Indstillinger

Dagens kost

Her er dit værktøj til at registrere dine daglige måltider og se hvor mange kalorier de indeholder. Du får hjælp fra SoSu-livs felter og farver til at sammensætte nogle sunde og mættende måltider, der passer lige til dit daglige behov.

Du kan indtaste alle dine måltider fra morgen til aften, ved hjælp af feltlistens mange varer. Dine anbefalede felter og farver fortæller dig, hvor mange felter og kalorier du kan spise i løbet af dagen, og hvordan du bør sammensætte måltidet. På den måde kan du holde øje med, om dine daglige måltider matcher SoSu-livs personlige anbefaling til dig, og nemt holde din vægt eller tabe dig.

God fornøjelse.

August 2012 | **Fredag d. 17** Åben kalender ? Vis felter som kJ (kilojoule) Om feltsystemet

Anbefalede felter: **40 felter** ?
Felter tilbage: **40 felter / 10000 kJ**
Bonusfelter: 0 felter

Protein	0		Mindst 6 felter
Fedt	0		Højst 13 felter
Sukker	0		Ikke for mange
Kulhydrat	0		Resten af felterne
Alkohol	0		

Morgenmad	0 felter (0 kJ)
Formiddag	0 felter (0 kJ)
Frokost	0 felter (0 kJ)
Eftermiddag	0 felter (0 kJ)
Aftensmad	0 felter (0 kJ)

Feltlisten | Manuel indtastning | Søg

Tilføj til: Sen aften ?
Vælg kategori: Favoritliste

Tilføj til listen Tilføj

SoSu-liv

MIN SIDE | KOLLEGAER | KOST | MOTION | RYGNING | VELVÆRE

Kost > Kogebog > Aftensmad > **Mexi-gryde med røde bønner, oksekød og friske tomater**

Mexi-gryde med røde bønner, oksekød og friske tomater

4 personer

1 Ingredienser 2 Indkøbsliste 3 Sådan gør du

400 g. hakket oksekød
250 g. kogte røde bønner
2 stk. friske tomater
1/4 frisk chili
1 tsk. olivenolie
1 stk. alm. løg
20 g mørk chokolade
½ dl tomatpuré
1 1/4 dl vand

Antal felter pr. portion:
5,4

3,1 | 0,9 | 0 | 1,4 | 0

Sværhedsgrad:

Tilberedningstid:
50 minutter

Arbejdstid:
20 minutter

Giv en karakter

Del på Facebook

Del denne artikel på Facebook
Klik her

Find opskrifter

Skriv dit søgeord i feltet og find hurtigt din opskrift

Søg

Send til en ven

Del denne side med en ven, kollega, familie **Klik her »**

Vidste du...

.. at du skal løbe i cirka en time for at forbrænde fire almindelige øl.

Afstemning

Går du op i at spise sundt?

- Ja
- Nogle gange

Aftensmad

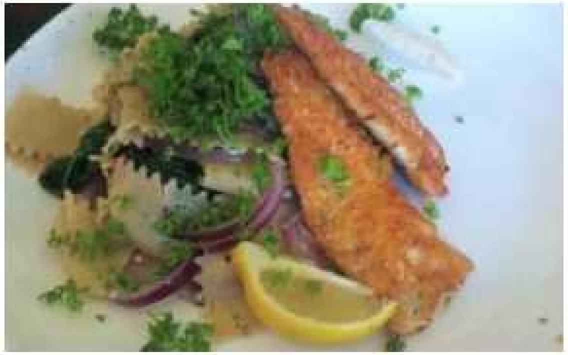

Parmesan panerede rødspættefiletter »

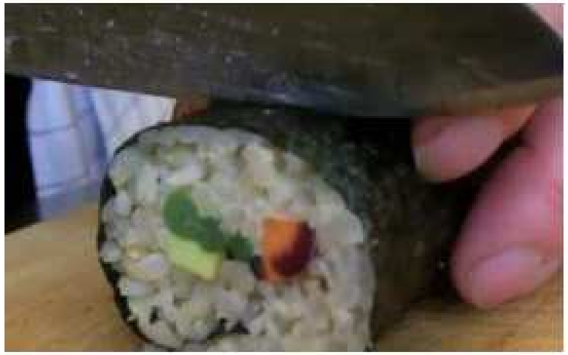

Grøntsags sushi - Makiruller »

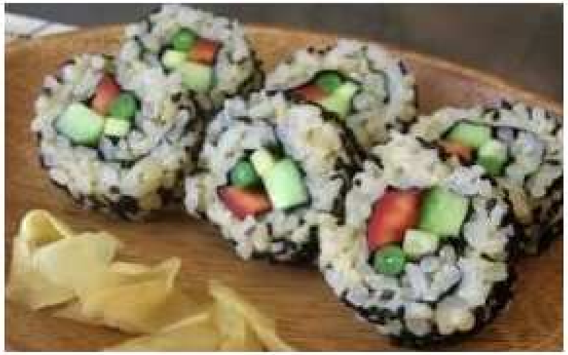

Grønsags sushi II – inside-out »

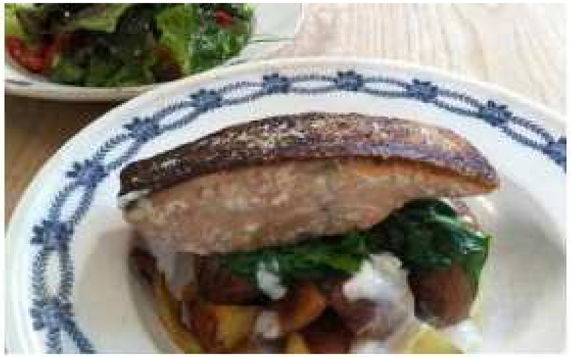

Stegt laks »

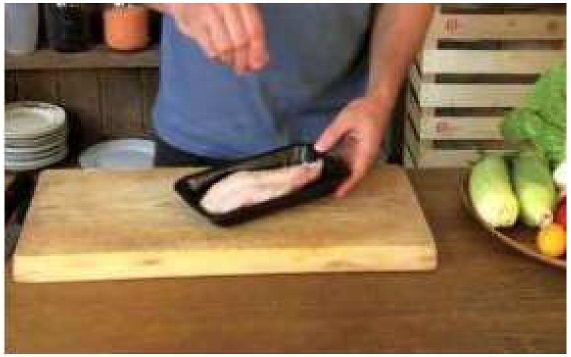

Stegt kyllingebryst »

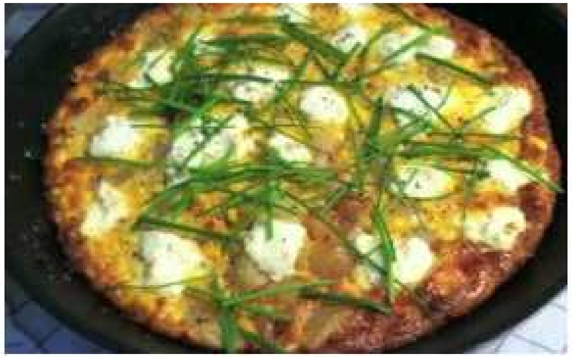

Æggekage med majs, kartofler og gedeost »

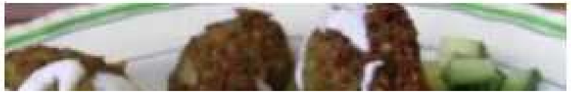

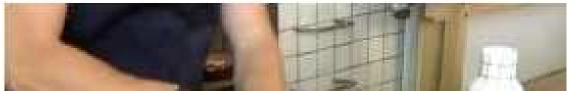

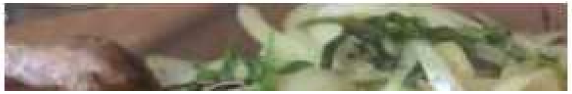

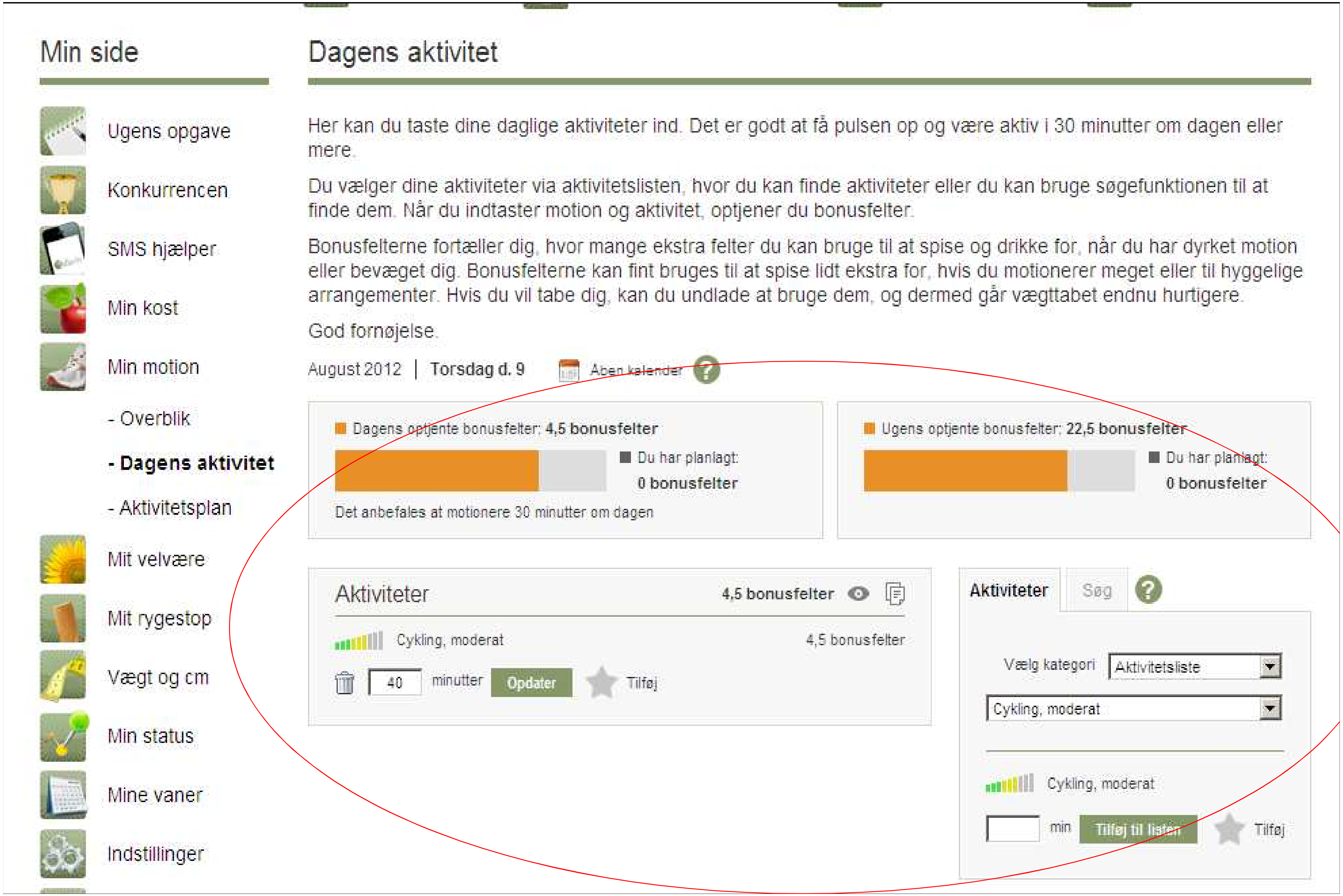
Min side
Ugens opgave
Konkurrencen
SMS hjælper
Min kost
Min motion
- Overblik
- Dagens aktivitet
- Aktivitetsplan
Mit velvære
Mit rygestop
Vægt og cm
Min status
Mine vaner
Indstillinger
Dagens aktivitet
Her kan du taste dine daglige aktiviteter ind. Det er godt at få pulsen op og være aktiv i 30 minutter om dagen eller mere.
Du vælger dine aktiviteter via aktivitetslisten, hvor du kan finde aktiviteter eller du kan bruge søgefunktionen til at finde dem. Når du indtaster motion og aktivitet, optjener du bonusfelter.
Bonusfelterne fortæller dig, hvor mange ekstra felter du kan bruge til at spise og drikke for, når du har dyrket motion eller bevæget dig. Bonusfelterne kan fint bruges til at spise lidt ekstra for, hvis du motionerer meget eller til hyggelige arrangementer. Hvis du vil tabe dig, kan du undlade at bruge dem, og dermed går vægttabet endnu hurtigere.
God fornøjelse.
August 2012 | Torsdag d. 9
Åben kalender
Dagens optjente bonusfelter: 4,5 bonusfelter
Du har planlagt:
0 bonusfelter
Det anbefales at motionere 30 minutter om dagen
Ugens optjente bonusfelter: 22,5 bonusfelter
Du har planlagt:
0 bonusfelter
Aktiviteter
4,5 bonusfelter
Cykling, moderat
4,5 bonusfelter
40
minutter
Opdater
Tilføj
Aktiviteter
Søg
Vælg kategori
Aktivitetsliste
Cykling, moderat
Cykling, moderat
min
Tilføj til listen
Tilføj

SoSu-liv

MIN SIDE | KOLLEGAER | KOST | MOTION | RYGNING | VELVÆRE

Motion > **Quiz**

Quiz

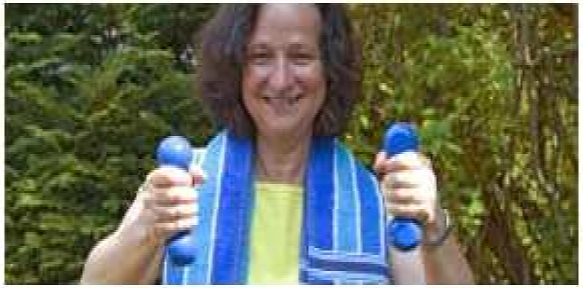

Træningstips »

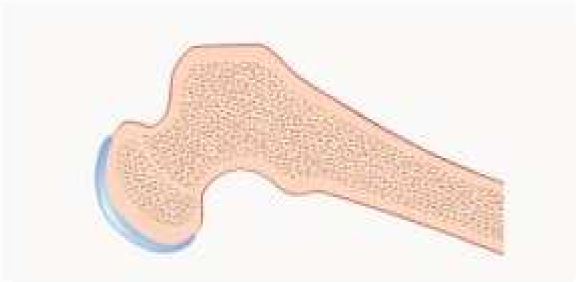

Knogleakademiet »

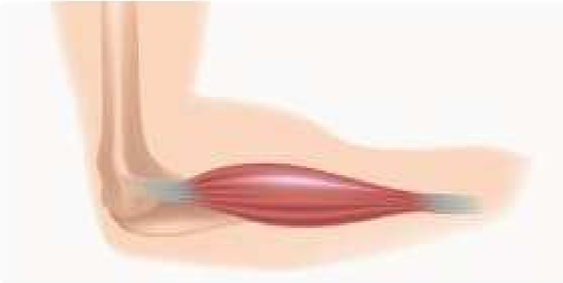

Hvor meget ved du om muskler? »

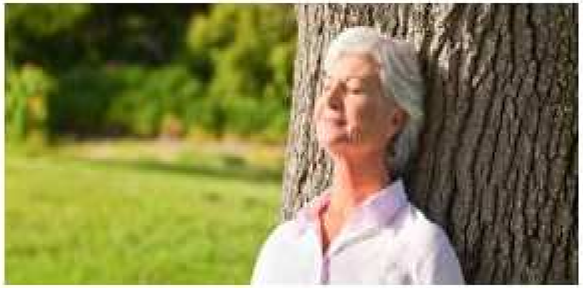

En dyb indånding »

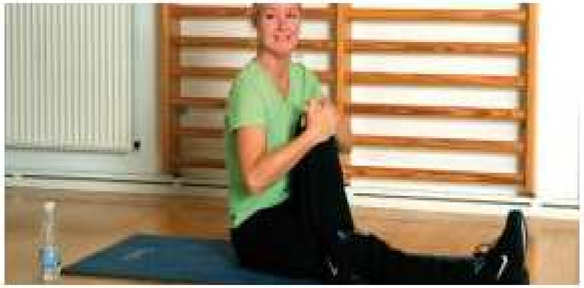

Kvinder og træning »

Min side

- Ugens opgave
- Konkurrencen
- SMS hjælper
- Min kost
- Min motion
- Mit velvære
- Mit rygestop
  - **- På vej**
  - - Rygestop
  - - Ikke ryger
- Vægt og cm
- Min status
- Mine vaner
- Indstillinger

På vej

Her kan du som ryger på en nem og overskuelig måde registrere det antal cigaretter, du ryger på en dag, samt finde frem til, om der er et mønster i din rygning. Du kan bruge dette redskab til at blive klar over, hvor meget du ryger, og om det er i bestemte situationer, eller i et bestemt humør du ryger mest.

Dette hjælper dig til at forstå dine egne rygevaner, og du kan bruge det til at finde frem til, om du ønsker at stoppe med at ryge.

Registrering af rygning ?

0

Se udvikling »

(Indtastningen nulstilles automatisk HVER dag kl. 23:59)

Fordele og ulemper ?

Fordele ved rygning:	0
Ulemper ved rygning:	0
Fordele ved rygestop:	0
Ulemper ved rygestop:	0

Gå til indtastning »

Rygemønster ?

Cigaretter røget i dag: 0

Gå til indtastning »

Bliv klar til rygestop

Få gode råd og vejledning til dit rygestop.

Læs mere »

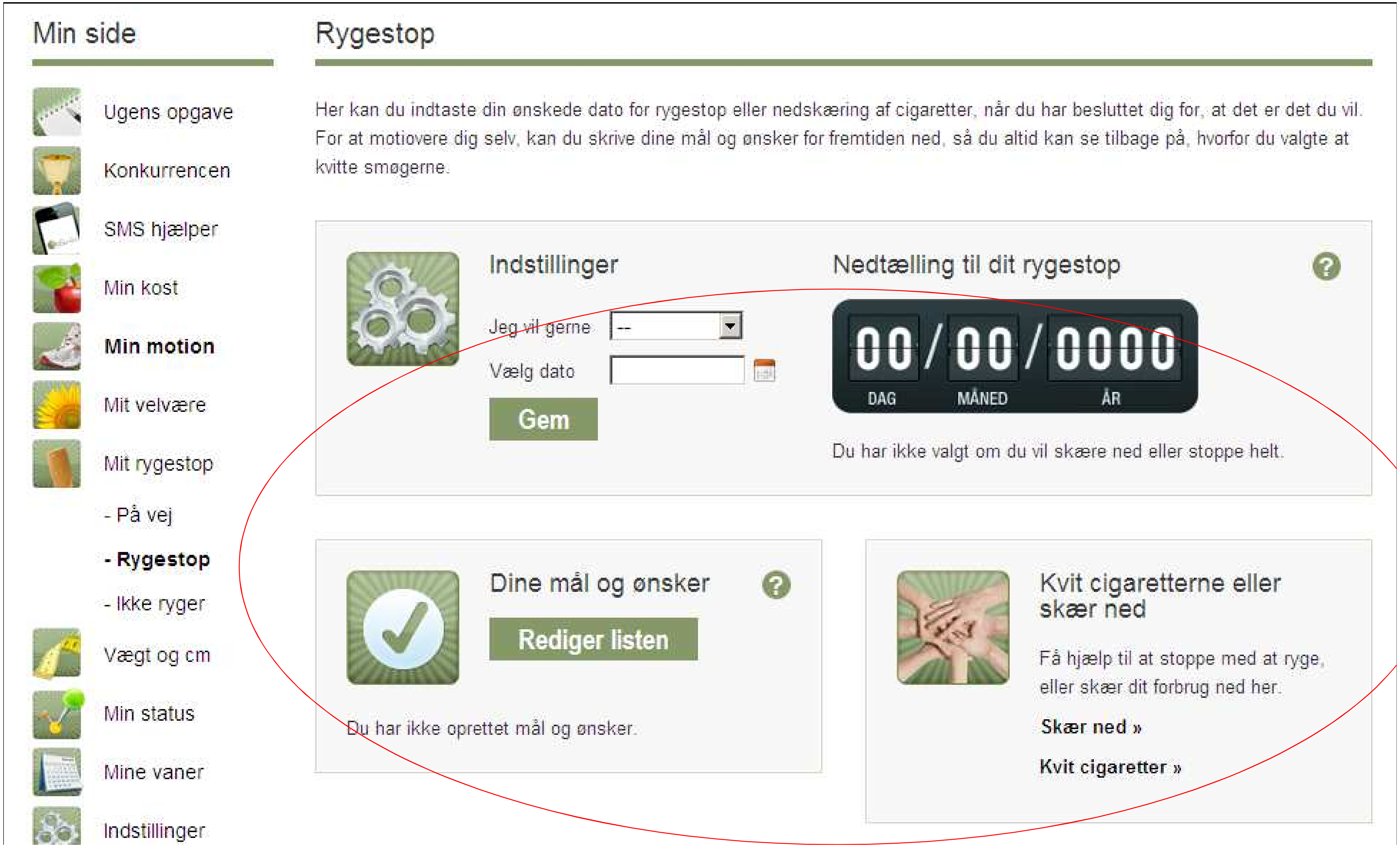
Min side
Ugens opgave
Konkurrencen
SMS hjælper
Min kost
Min motion
Mit velvære
Mit rygestop
- På vej
- Rygestop
- Ikke ryger
Vægt og cm
Min status
Mine vaner
Indstillinger
Rygestop
Her kan du indtaste din ønskede dato for rygestop eller nedskæring af cigaretter, når du har besluttet dig for, at det er det du vil. For at motiovere dig selv, kan du skrive dine mål og ønsker for fremtiden ned, så du altid kan se tilbage på, hvorfor du valgte at kvitte smøgerne.
Indstillinger
Jeg vil gerne
Vælg dato
Gem
Nedtælling til dit rygestop
00/00/0000
DAG
MÅNED
ÅR
Du har ikke valgt om du vil skære ned eller stoppe helt.
Dine mål og ønsker
Rediger listen
Du har ikke oprettet mål og ønsker.
Kvit cigaretterne eller skær ned
Få hjælp til at stoppe med at ryge, eller skær dit forbrug ned her.
Skær ned »
Kvit cigaretter »

Den store konkurrence: Danmarks bedste SoSu'ere

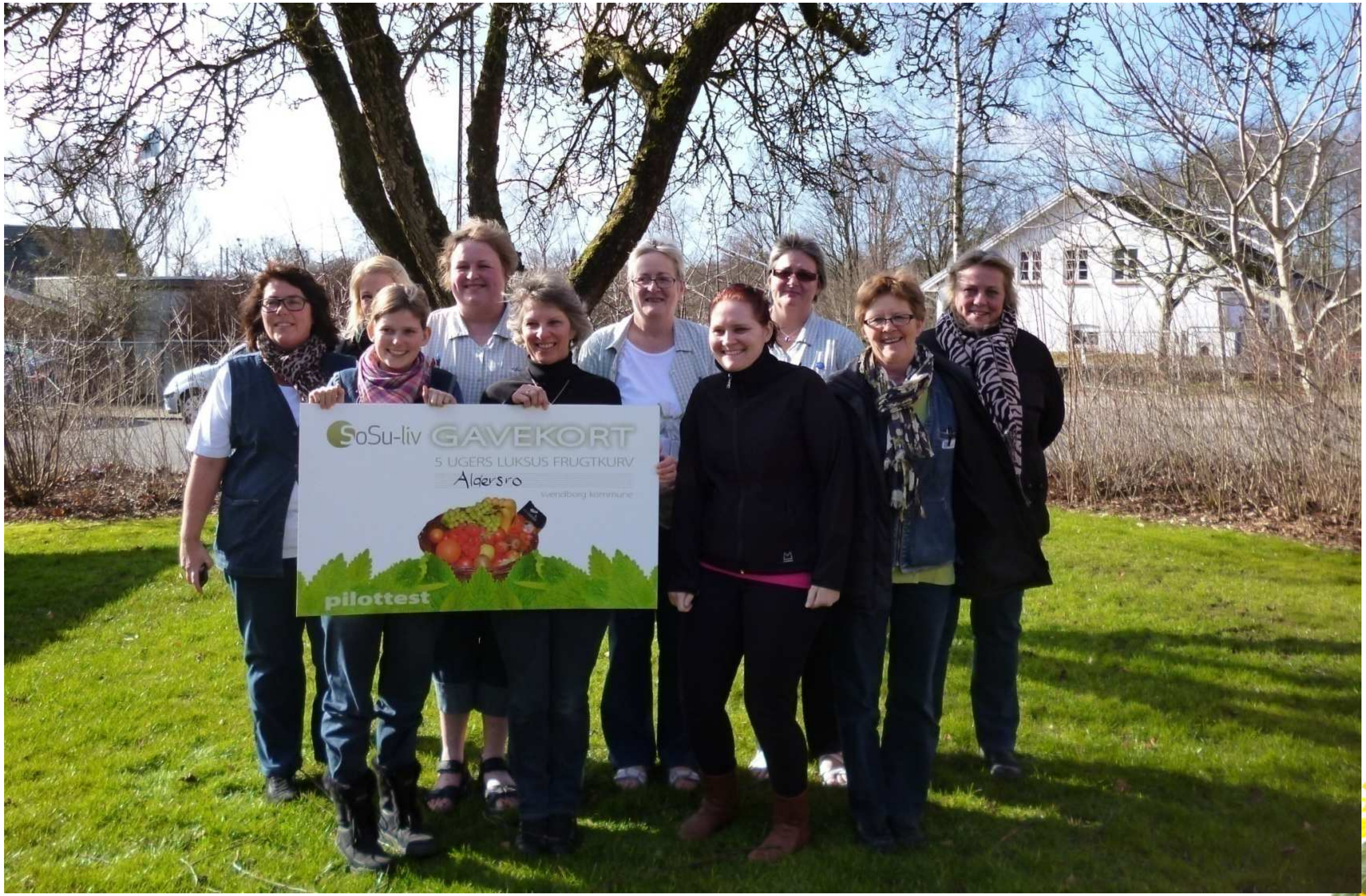

SoSu-liv

Den store konkurrence: Danmarks sundeste plejecenter

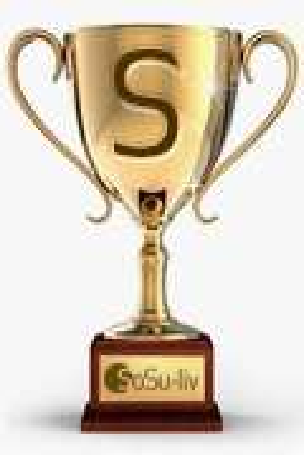

Dit hold: Alle tilmeldte kollegaer på din arbejdsplads

Varighed: 16 uger

Disciplin: Aktiviteter, øvelser og opgaver inde for kost, motion, rygning og velvære

Point: Alle aktiviteter giver point
- på hjemmesiden og i den fysiske verden

Holdets point: Gns. point/medarbejder

Vinder: Flest point i gns./medarbejder efter 16 uger

Præmie: En aften på plejecenteret med Karen Marie Lillelund
- med op til 100 gæster

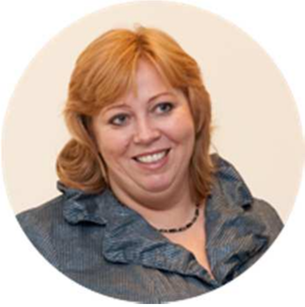

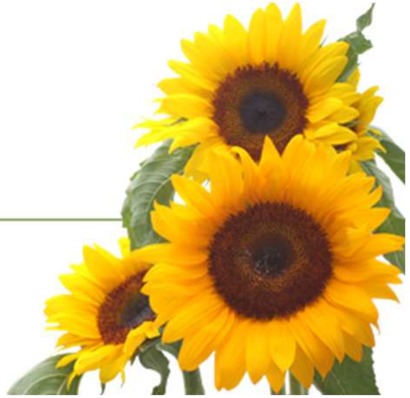

Løbende konkurrence: Månedens hold

Opmuntringer undervejs

- Én gang om måneden kåres månedens hold (september, oktober osv.)
- Holdets point laves om til lodder

Dit holds lodder indgår i lodtrækning

- Ét hold vinder lodtrækningen
- Vinderne får direkte besked og offentliggøres på hjemmesiden

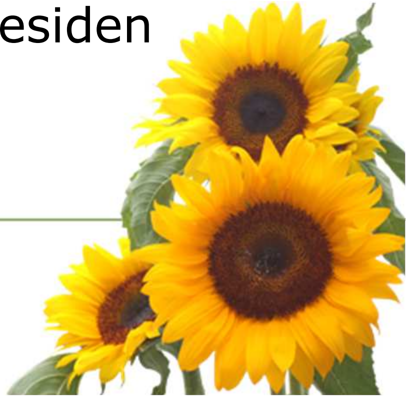

Følg stillingen i konkurrencen

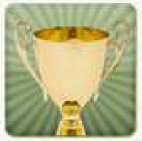

SoSu-liv konkurrencen

1. Finsensvej (Frederiksb (0)
2. Hospitalet (Frederiksbe (0)
3. Howitzvej (Frederiksbe (0)

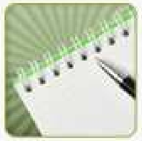

Månedens hold

Tallet i parantes angiver antal lodder til lodtrækningen.

1. Finsensvej (Frederiksb (0)
2. Hospitalet (Frederiksbe (0)
3. Howitzvej (Frederiksbe (0)

Læs om de spændende præmier I kan vinde

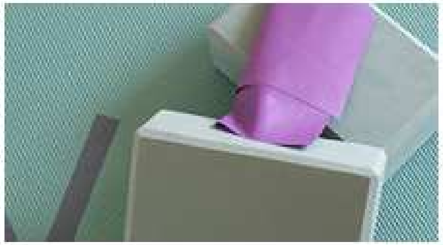

September-holdet

En taske med træningselastik og øvelseshæfte, så I kan få musklerne til at slappe af.

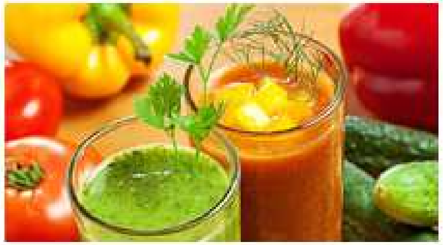

Oktober-holdet

Et besøg af en professionel bartender, så I kan få serviceret sunde, frisklavede smoothies.

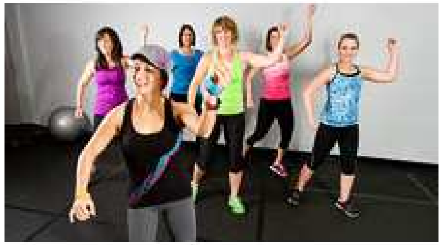

November-holdet

En svingende oplevelse, hvor I bliver undervist i Zumba.

SoSu-liv

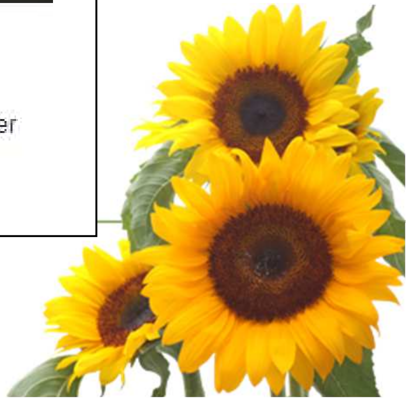

SoSu-liv som et forskningsprojekt

- SoSu-liv projektet er anmeldt til
    - Den Videnskabsetiske Komité
    - Datatilsynet

Du skal give dit samtykke til din deltagelse efter informationsmødet

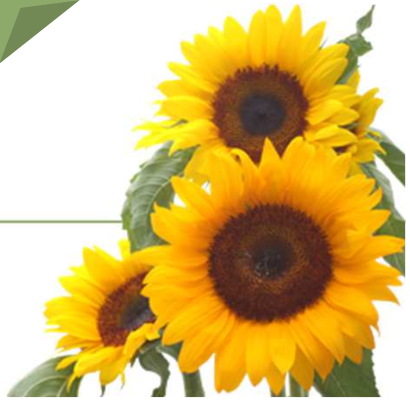

Forskning med lodtrækning

- Projektets deltagere er delt i 2 grupper
  - nogle skal bruge SoSu-liv
  - andre skal **<u>ikke</u>** bruge SoSu-liv (kontrol)

Vi interesserer os for forskellen i resultaterne efter 9 måneder

I skal bruge SoSu-liv i de næste 9 måneder

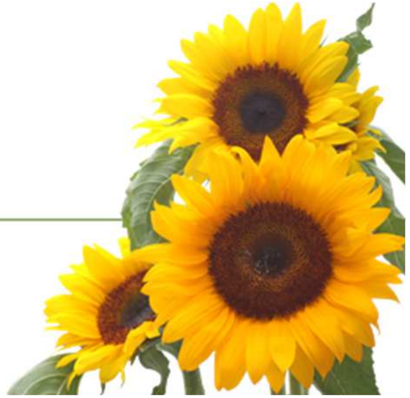

SoSu-liv kommuner

Halsnæs Kommune

Hvidovre

FREDERIKSBERG KOMMUNE

Sønderborg

HILLERØD KOMMUNE

Ishøj Kommune

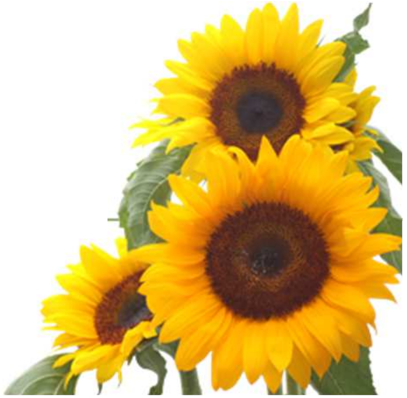

1. Trin: Elektronisk spørgeskema på hjemmesiden

- Du skal svare på et spørgeskema om dig selv, dit helbred, dine vaner og din trivsel

- Udfyld skemaet derhjemme, inden du kommer til helbreds-undersøgelsen. Det tager ca. 15-20 minutter

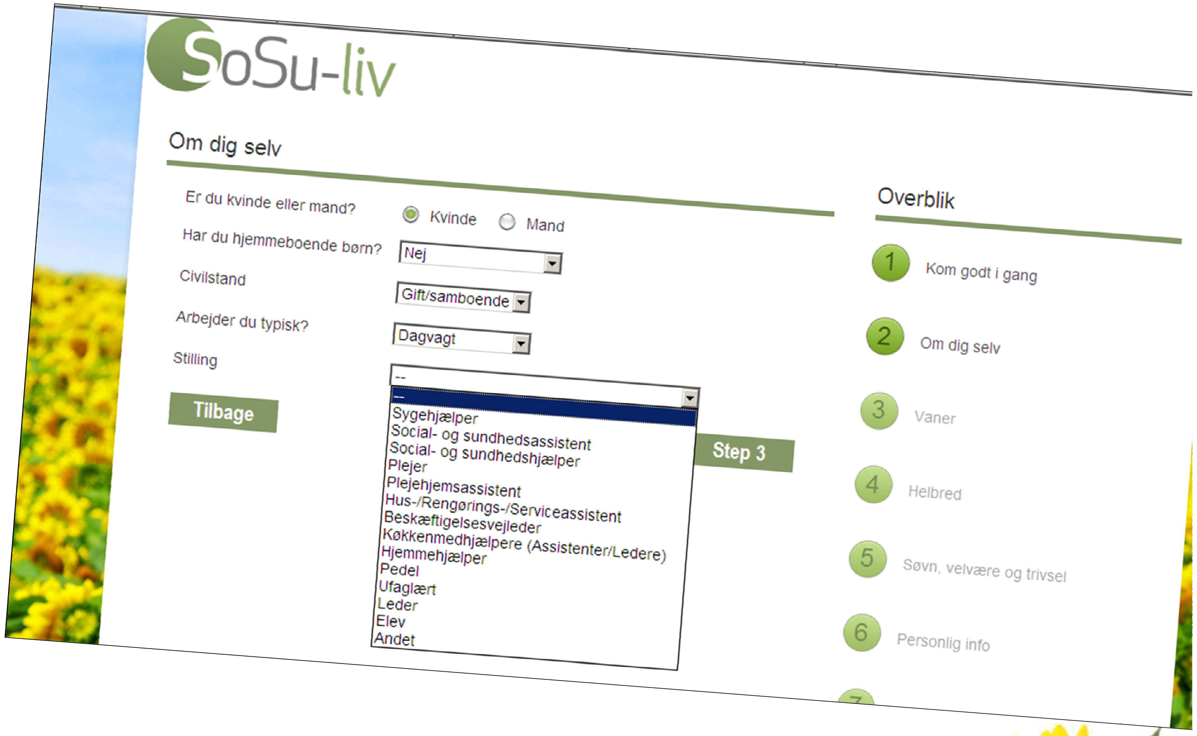

HOTLINE
44 39 34 12

SoSu-liv

Spørgeskema på www.sosu-liv.dk

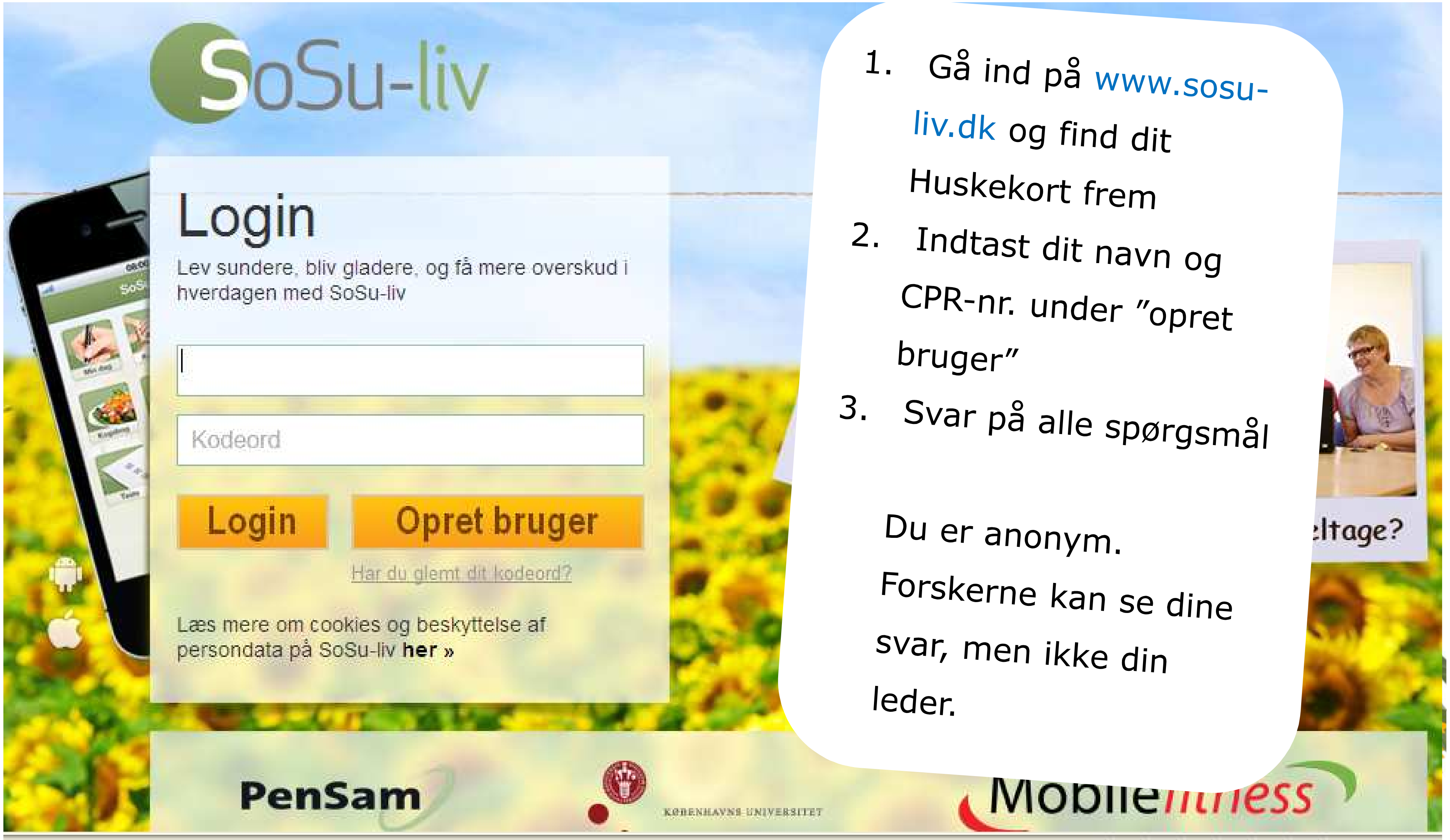

1. Gå ind på www.sosu-liv.dk og find dit Huskekort frem
2. Indtast dit navn og CPR-nr. under "opret bruger"
3. Svar på alle spørgsmål

Du er anonym.
Forskerne kan se dine svar, men ikke din leder.

2. Trin: Fysisk helbredsundersøgelse (Uge 0, 16, 38)

Vi måler:

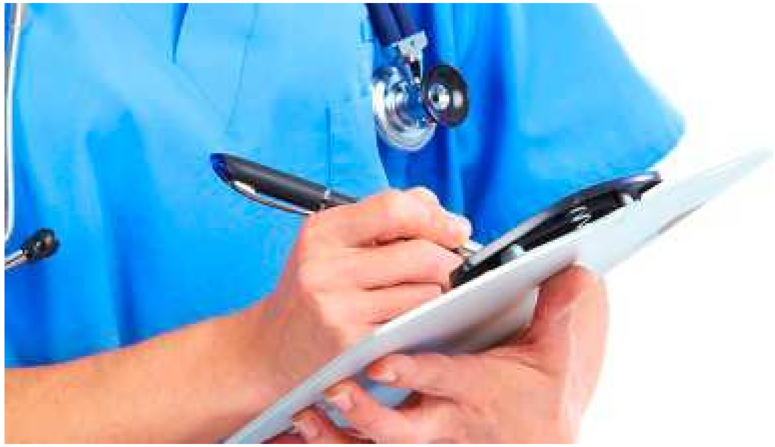

- Højde
- Vægt
- Talje- og hofteomkreds
- Kropsfedt-procent
- Blodtryk
- Kolesterol (prik)
- Blodsukker (prik)

- Det tager ca. 15 minutter

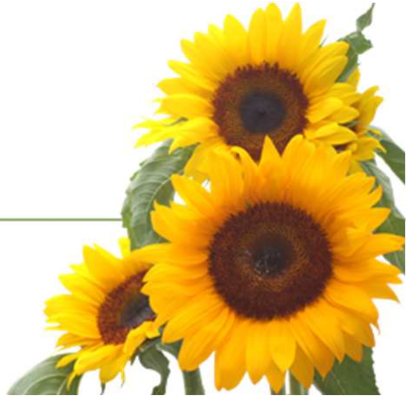

Fysisk helbredsundersøgelse

- Selve helbredstjekket foregår her på stedet
- Du bliver målt **i enerum** – **i undertøj** af en SoSu-liv medarbejder
- Vi anbefaler, at du ikke har spist de sidste 3 timer
- Du får dine tal på papir med det samme
- Gem dine tal til næste undersøgelse om ca. 16 uger

HOLD ØJE
med tid og sted
for helbredsundersøgelse

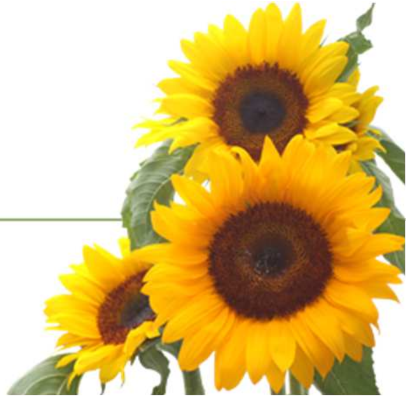

Trin 3. Udvælg ét indsatsområde (løfte)

- Ud fra dine tal i spørgeskemaet og fra helbredsundersøgelsen vil SoSu-liv give dig én anbefaling om enten at:
  - tabe dig
  - spise sundere
  - komme i form
  - blive stærkere
  - skære ned på cigaretter
  - stoppe med at ryge
  - fastholde en sund livsstil

KOM i PC CAFÉEN – Vi hjælper i gang

- Du vælger ét område, som du vil arbejde med de næste 16 uger. Vi kalder det "dit løfte"

- Efter 16 uger kan du evt. vælge et nyt løfte

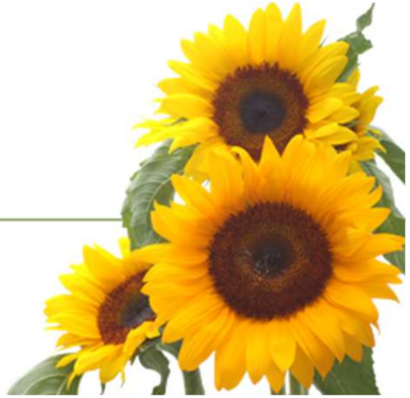

SoSu-liv

Så er du i gang på SoSu-Liv….

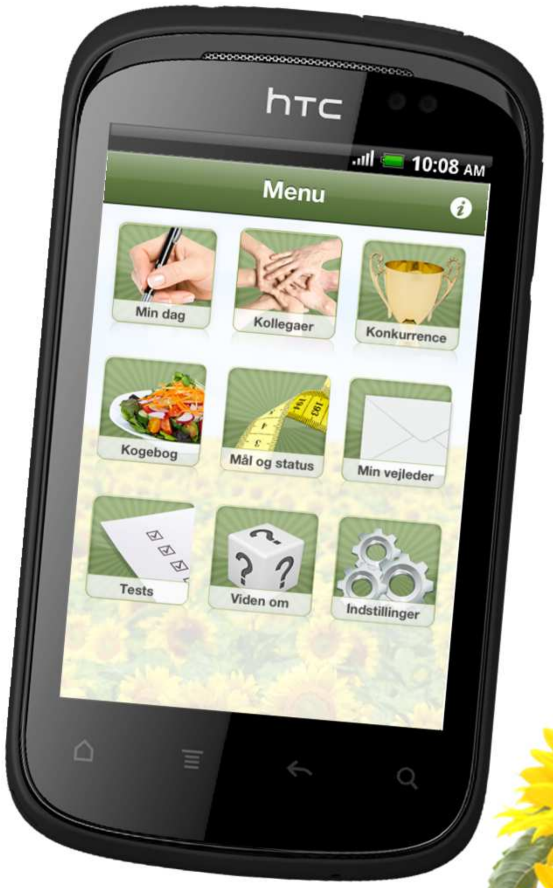

SoSu-liv

Få hjælp og støtte fra projektet

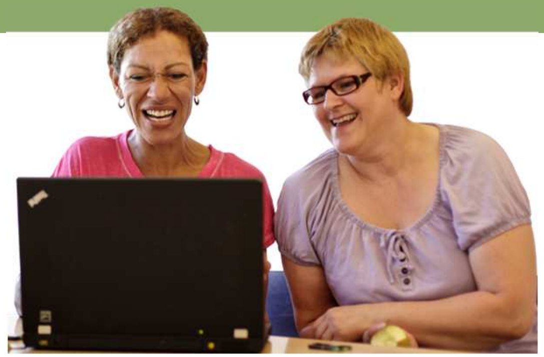

- Udfyldelse af spørgeskema, brug af mobiltelefon eller hjemmeside
  - Ring på 44 39 34 12 eller mail til sosuliv@pensam.dk

- Teknisk Support
  - Ring på 22 78 25 28 til Claus

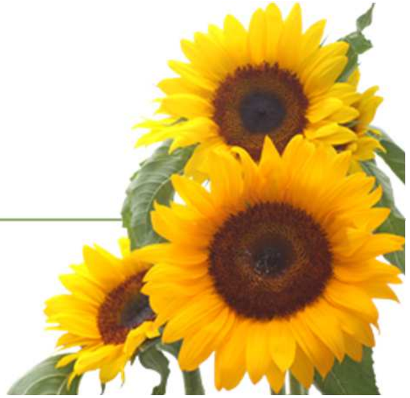

Næste vigtige skridt

1. Find mappen frem
   - Underskriv SAMTYKKE-erklæringen
   - Aflever erklæringen til din leder og få dit personlige huskekort
2. Hjemme
   - Log på første gang
   - Udfyld spørgeskemaet
3. På arbejdet
   - Helbredstjek

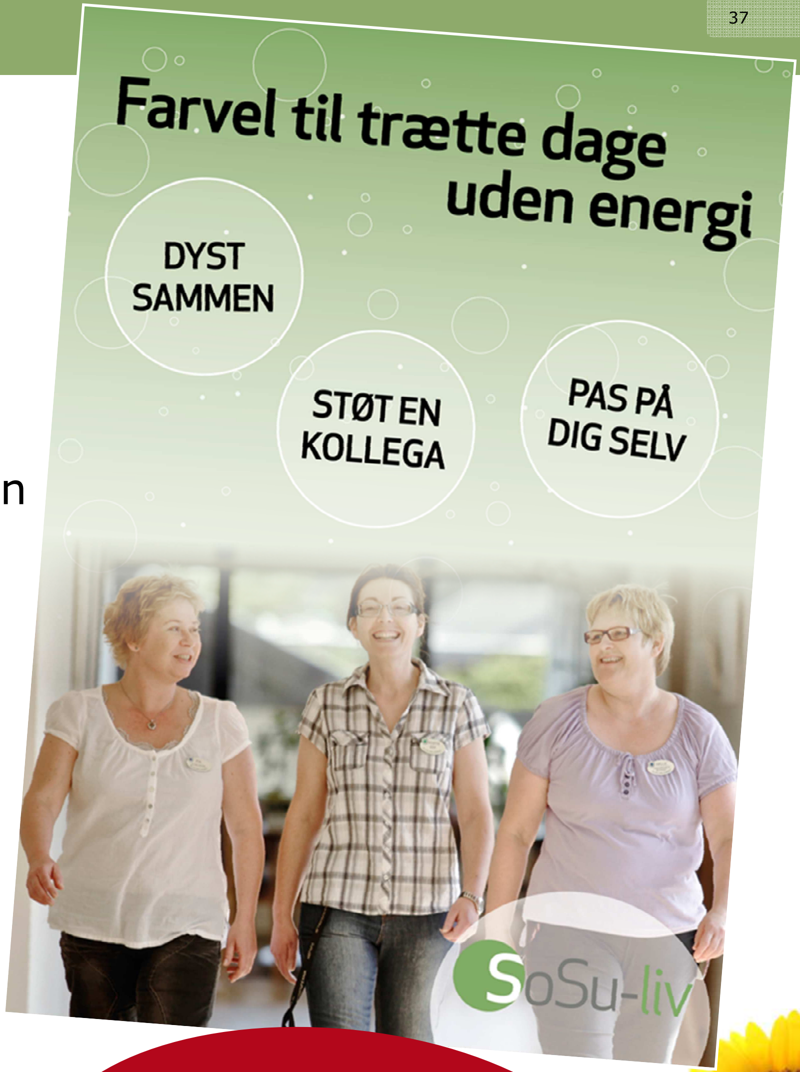

Vi ses til HELBREDSUNDERSØGELSE

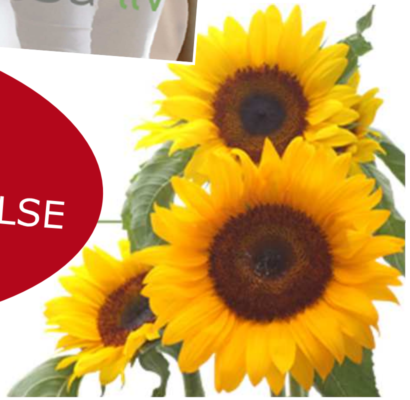

SoSu-liv
